# Geospatial Analysis of Sodium and Potassium Intake: A Swiss Population-Based Study

**DOI:** 10.3390/nu13061798

**Published:** 2021-05-25

**Authors:** David De Ridder, Fabiën N. Belle, Pedro Marques-Vidal, Belén Ponte, Murielle Bochud, Silvia Stringhini, Stéphane Joost, Idris Guessous

**Affiliations:** 1 Laboratory of Geographic Information Systems (LASIG), School of Architecture, Civil and Environmental Engineering (ENAC), École Polytechnique Fédérale de Lausanne (EPFL), 1015 Lausanne, Switzerland; david.deridder@unige.ch (D.D.R.); stephane.joost@epfl.ch (S.J.); 2 Unit of Population Epidemiology, Division of Primary Care Medicine, Department of Primary Care Medicine, Geneva University Hospitals, 1205 Geneva, Switzerland; Silvia.Stringhini@hcuge.ch; 3 Faculty of Medicine, University of Geneva, 1205 Geneva, Switzerland; 4 Group of Geographic Information Research and Analysis in Population Health (GIRAPH), 1205 Geneva, Switzerland; Pedro-Manuel.Marques-Vidal@chuv.ch; 5 Institute of Social and Preventive Medicine (ISPM), University of Bern, 3012 Bern, Switzerland; fabien.belle@ispm.unibe.ch; 6 Center for Primary Care and Public Health (Unisanté), University of Lausanne, 1010 Lausanne, Switzerland; murielle.bochud@uniste.ch; 7 Department of Medicine, Internal Medicine, Lausanne University Hospital, 1011 Lausanne, Switzerland; 8 Service of Nephrology and Hypertension, University Hospital Geneva, 1205 Geneva, Switzerland; belen.ponte@hcuge.ch; 9 La Source, School of Nursing, University of Applied Sciences and Arts Western Switzerland (HES-SO), 1004 Lausanne, Switzerland

**Keywords:** geospatial analysis, sodium, potassium, GWR, social determinants of health, GIS, spatial clustering

## Abstract

Inadequate sodium and potassium dietary intakes are associated with major, yet preventable, health consequences. Local public health interventions can be facilitated and informed by fine-scale geospatial analyses. In this study, we assess the existence of spatial clustering (i.e., an unusual concentration of individuals with a specific outcome in space) of estimated sodium (Na), potassium (K) intakes, and Na:K ratio in the Bus Santé 1992–2018 annual population-based surveys, including 22,495 participants aged 20–74 years, residing in the canton of Geneva, using the local Moran’s *I* spatial statistics. We also investigate whether socio-demographic and food environment characteristics are associated with identified spatial clustering, using both global ordinary least squares (OLS) and local geographically weighted regression (GWR) modeling. We identified clear spatial clustering of Na:K ratio, Na, and K intakes. The GWR outperformed the OLS models and revealed spatial variations in the associations between explanatory and outcome variables. Older age, being a woman, higher education, and having a lower access to supermarkets were associated with higher Na:K ratio, while the opposite was seen for having the Swiss nationality. Socio-demographic characteristics explained a major part of the identified clusters. Socio-demographic and food environment characteristics significantly differed between individuals in spatial clusters of high and low Na:K ratio, Na, and K intakes. These findings could guide prioritized place-based interventions tailored to the characteristics of the identified populations.

## 1. Introduction

Elevated blood pressure and hypertension are major, yet preventable, risk factors for cardiovascular diseases and mortality, contributing to 49% of all coronary heart disease and 62% of all stroke events, and are leading causes of morbidity and mortality worldwide [1,2,3,4,5]. Diets low in sodium (Na) and high in potassium (K) reduce the risk of hypertension and related diseases [3,4,6,7,8]. Findings from past studies suggest that the ratio of the two nutrients (Na:K ratio) is an even more important risk factor for cardiovascular disease and mortality and, therefore, Na and K intakes should be examined jointly [9,10]. The World Health Organization (WHO) recommends restricting Na intake to 2 g/day (equivalent to 5 g salt/day), increasing K intake to 3.5 g/day [11] and having a Na:K intake ratio of around 1 for optimal health [12]. Nevertheless, in most countries around the world, populations are consuming much more Na and less K than recommended by the WHO guidelines [11,13,14]. Salt reduction strategies are considered essential and cost-effectives measures to improve public health [15,16,17]. Accordingly, various public health and policy interventions have been implemented internationally and nationally to promote diets low in Na and to increase attention to the risks associated with inadequate Na and K intakes [18]. In Switzerland, for example, where adults consume around 9 g per day of salt, the Salt Strategy, which has been in place since 2008, aims to reduce the population’s salt consumption, gradually and on a voluntary basis, to reach a consumption of less than 8 g per day in the medium term and less than 5 g per person per day in the long term [19].

Several studies have pointed out the existence for socioeconomic and geographic gradients in salt intake, with socioeconomically disadvantaged populations having higher intake of Na and lower K intake [20,21,22]. These populations are more likely to rely on cheap, processed foods, which are often high in salt [20]. Numerous studies have also assessed the association between the local food environment and dietary behaviors, mainly in the context of the obesity epidemic [23,24]. However, until now, no study has jointly examined the relations between socio-demographic and food environment characteristics and Na:K ratio, Na, and K intakes.

In recent decades, spatial-based information and spatial analysis methods have been increasingly used in epidemiological research to explore and examine the role of “place” as a contextual factor for different health risk factors [25,26]. These spatial approaches offer exploratory and explanatory insights beyond conventional epidemiological association studies, which implicitly rely on the assumption that associations are invariant across space. Most existing studies considering spatial variations are conducted using large administrative units by aggregating individual-level data, which may alter the original signal or not coincide with an adequate scale for the implementation of local policies or public health interventions aimed at promoting a diet low in Na and high in K. Spatial statistics and clustering methods allow determining whether observations in a study area with a specific outcome are randomly distributed or unusually concentrated in space. In case of spatial clustering, factors that may explain spatial dependence may be investigated to provide insights into their underlying causes [26]. In this study, we aimed to (i) verify the existence of spatial dependence of Na:K ratio, Na, and K intakes in a large population-based study and (ii) investigate whether socio-demographic and residential food environment factors are associated with the identified spatial clusters.

## 2. Materials and Methods

### 2.1. Study Design and Sample

Data were collected through the Bus Santé study, a continuing population-based study in the canton of Geneva, Switzerland, with approximately 500,000 inhabitants in 2021, monitoring health and cardiovascular risk factors. Independent samples of residents were subjected to health examination surveys since 1993. The recruitment procedure has been described in details in previous studies [27,28]. The average participation rate was 59% (SD ± 7.4%) and ranged from 48% (2008) to 75% (2017). The geographic coordinates of the residential address of the Bus Santé participants were used for spatial analyses. The Bus Santé study was approved by the Cantonal Research Ethics Commission of Geneva, Switzerland (PB_2016-00363).

### 2.2. Food Frequency Questionnaire

Na and K intakes were assessed using a self-administered, semiquantitative food frequency questionnaire (FFQ) originally developed and validated against 24-h dietary recalls. The same FFQ was used throughout the entire study period (1993–2018). This FFQ has been described in details elsewhere [25,29]. Daily Na and K intakes estimated using this FFQ have been used in a previous study [30]. Briefly, Belle et al. converted food portions into micronutrients based on two different food composition datasets: the French Information Center on Food Quality and the Swiss Food Composition Database of the Federal Food Safety and Veterinary Office [31,32]. K intake was calculated by summing up the content of each FFQ item. We calculated Na intake using equations developed specifically for this FFQ for males and females separately [13,30]:

(1) $$Males:\;\frac{\left(8.2+0.38*Na\;\left(g/day\right)\;from\;FFQ\right)}{2.54}\;$$

(2) $$Females:\;\frac{\left(4.55+0.67*Na\;\left(g/day\right)\;from\;FFQ\right)}{2.54}$$

These equations are based on calibrations on total salt intake from 24-h urine collections in a validation study that included 100 healthy people [13,30]. Salt intake was converted into Na intake, 1 g Na being equal to 2.54 g of salt (NaCl). The Na:K ratio corresponds to the estimated Na intake (g/day) divided by the estimated K intake (g/day) [30].

### 2.3. Individual-Level Socio-Demographic Characteristics

Educational attainment was categorized into three categories: tertiary education, secondary education, and primary education. Occupation was categorized into four categories: high (professional and intermediate professions), medium (non-manual occupations), low (manual or lower occupations), and not working (unemployed, retired, or stay-at-home participants, for whom data on their last occupied job was not available) [33]. Civil status was dichotomized as being married/cohabiting or not; age was defined as a continuous variable; nationality was dichotomized as having Swiss nationality or not.

### 2.4. Neighborhood Food Environment

We obtained the dataset of all registered companies in the canton of Geneva, compiled by the “Répertoire des entreprises de Genève” (REG) [34] and made available by the “Système d’information du territoire à Genève” (SITG) [35]. Yearly listings of all the companies registered in the canton were available from 2003 to 2018, except 2006 and 2007, and contained designations that allowed us to disaggregate by outlet type, including supermarket, grocery, and convenience stores. Geographic coordinates of each amenity were provided in the dataset.

For each participant, we assessed the density of each food outlet category through accessibility analyses. To consider the evolution in the built environment over the study period (1993–2018), we matched the participation year of each participant to the closest year available in the food outlet dataset. The number of food outlets by category and year is described in Table S1.

For each participant and each food outlet category, we computed the density of food outlets in the neighborhood. A linear decay function with a bandwidth of 800 m was used to account for decreasing attractivity as the distance from home increased [36,37]. The street network of the canton of Geneva was obtained using the OSMnx Python package [38]. The study participants and food outlets were snapped (50 m maximum snapping distance) to the nearest segment of the street network, and the Pandana Python package [39] was used to perform the accessibility analyses. We specifically defined neighborhoods as an 800 m street network distance for three reasons. First, distances between 400 m and 800 m are often used as acceptable walking distances [36,37,40]. Secondly, street network buffers, in comparison to circular buffers, better capture human mobility patterns because they account for the constrains imposed by the street connectivity and impermeable barriers [41]. Finally, the 800 m distance corresponds to the distance of maximum global spatial autocorrelation for the Na:K ratio (see Section 3.3.1). Maps of the spatial distribution of the density of the three different food outlets are presented in Figure S1.

### 2.5. Additional Area-Level Explanatory Variable

Since individual income data was unavailable, we used the yearly area-level median household income for the years from 2005 to 2016, assigned to each individual based on their year of participation (i.e., 1993–2004 assigned to 2005 and 2017 and 2018 assigned to 2016) and their corresponding statistical subsector in the Geneva (GIRECs)-neighborhood definition by the State of Geneva (Office Cantonal de la Statistique, www.ge.ch/statistique (accessed on 28 November 2020)).

### 2.6. Exclusion Criteria

We excluded participants that were not located within the canton of Geneva (*n* = 17, 0.06%). We excluded participants outside the 20–74 age range (*n* = 69, 0.3%) and those with missing data for civil status (*n* = 31, 0.1%), occupation (*n* = 164, 0.7%), educational attainment (*n* = 279, 1.1%), nationality *(n* = 92, 0.4%), Na and K intakes (*n* = 1, 0.004%), and neighborhood median household income (*n* = 332, 1.4%). We also excluded 1544 participants with extreme dietary intake (<850 kcal or >4500 kcal per day) [42]. A total of 22,495 individuals remained and were used for the analyses.

### 2.7. Regression Modelling

#### 2.7.1. Ordinary Least Squares Regression Modelling

We first estimated ordinary least squares (OLS) models of Na:K ratio, Na intake. and K intake in relation to neighborhood environment and socio-demographics characteristics. Three models were estimated for each outcome variable: (i) model 1 included neighborhood food environment variables, model 2 included individual-level socio-demographic characteristics and the neighborhood median household income, and model 3 included model 1 + model 2. All models were adjusted for total energy intake and year of participation.

#### 2.7.2. Geographically Weighted Regression Modelling

Our goal was to investigate associations with socioeconomic and residential food environment factors and analyze how the intensity and significance of these associations varied over the study area. In the state of Geneva, socio-demographic factors [43] and access to food outlets vary greatly across neighborhoods and municipalities (Figure S1). OLS may be inappropriate when observations are not spatially independent, which can be assessed by the presence of statistically significant spatial autocorrelation in the residuals [44]. Therefore, we used a geographically weighted regression (GWR), which accounts for spatial dependence in the observations. Indeed, this method performs a separate local regression model at each location, borrowing information from surrounding locations (i.e., the neighbors of any participant) [45]. This technique has been shown to improve model fit and reduce spatial autocorrelation in the residuals compared to OLS regression models [46,47].

We used the Python package PySAL [46,48] for the GWR model implementation. In this model, a circular kernel function is used to calculate a local regression at each location. We used a fixed Gaussian kernel function and an Akaike information criterion (AICc) optimized bandwidth. This kernel function allows to place more weight on nearby observations. Finally, spatial autocorrelation in the residuals was evaluated for both OLS and GWR models using the PySAL package in Python, where neighbors were defined based on a fixed-distance band of 800 m for Na:K ratio and Na intake and a fixed-distance band of 400 m for K intake based on the spatial scale determination (See Section 3.3.1). Given the high number of results outputted by GWR analyses, we only presented visualization of parameters estimates and *t*-values for the Na:K ratio adjusted with GWR model 3 (Figure S3).

### 2.8. Spatial Analysis

#### 2.8.1. Global Spatial Autocorrelation

Using the Python spatial analysis library PySAL 2.3, we assessed global spatial autocorrelation in Na and K intakes with the Global Moran’s *I* (GMI) statistic [49]. For the GMI calculations, we specified a fixed distance band spatial weights matrix: individuals within the distance band are given the same weight, while those outside the distance band are given a weight of zero [50]. The spatial weights matrix was row-standardized; this technique is used particularly with binary weighting strategies to create proportional weights in cases where individuals have an unequal number of neighbors [49]. The statistical significance was assessed with a Monte Carlo procedure, using a sample of 999 permutations [51]. The null hypothesis was that the variable is distributed randomly. Moran’s I values range between −1 and 1: a GMI close to −1 indicates spatial dispersion, while a GMI close to 1 indicates spatial clustering. A Moran’s I value near 0 indicates an absence of spatial dependence. To determine the distance corresponding to the maximum spatial autocorrelation in each outcome variable, subsequent GMI statistics were calculated at incremental fixed distance bands of 200 m, 400 m, 600 m, 800 m, and 1000 m. We selected the distance at which the statistically significant z-score peaked (i.e., the distance at which the spatial processes promoting clustering are the most pronounced) [52,53].

#### 2.8.2. Local Spatial Autocorrelation

Global measures of spatial dependence allow identifying whether spatial autocorrelation takes place in the study area but are unable to detect local clusters of high or low values that may exist. Additionally, spatial processes occurring in only parts of the study area can be missed [26]. We used the univariate Local Moran’s *I* statistic [51], a widely used method for evaluating local spatial autocorrelation and identifying local spatial clusters of both high and low Na:K ratio, Na, and K intakes. When calculating this statistic, we used the same row-standardized fixed distance band weight matrix used for the global spatial autocorrelation analysis. A significant positive local Moran’s *I* indicates that the individual under study has a similarly high or low value as its neighbors, forming two classes: the high-high class, with individuals showing a high value surrounded by neighbors with high values, and the low-low class, with individuals showing low values surrounded by neighbors with low values [51].

A significant negative local Moran’s *I* indicates outliers (i.e., individuals that differ from their neighbors), forming two additional classes: the high-low class (individuals with high value and low value neighbors) and the low-high class (individuals with low value and high value neighbors). Statistical significance was evaluated using a Monte-Carlo procedure, using a sample of 999 permutations. Values with a *p*-value below the 0.05 threshold were considered statistically significant.

Socio-demographic and food environment characteristics may be significant determinants of Na and K intakes and may potentially explain the presence of local clustering over the study area. Therefore, the Local Moran’s *I* statistic was calculated and mapped for each unadjusted outcome variable and for the residuals of models 1, 2, and 3 of each outcome variable. Local clusters persisting in the residuals correspond to spatial clustering not explained by the included covariates [26]. Finally, we compared socio-demographic and food environment characteristics of the high-high and low-low individuals using radar plots.

## 3. Results

### 3.1. Descriptive Statistics

#### 3.1.1. Sample Characteristics

After excluding participants for missing data, 22,495 (92.2%) participants were retained. The mean age of the participants in the Bus Santé study (1993–2018) was 50.1 years (SD ± 12.1 years); women and men were equally represented. Socio-demographic and food environment characteristics of all included participants are presented in Table 1.

#### 3.1.2. Dietary Sources of Sodium and Potassium Intake

The main sources of Na were breads and cereals (31%), dairy products (20%), vegetables and vegetable dishes (13%), processed meat (9%), and fish (7%) (Figure S2A). The main sources of K were vegetables and vegetable dishes (18%), fruit and fruit juices (17%), breads and cereals (17%), dairy products (13%), meat (8%), and fish (5%) (Figure S2B).

### 3.2. Regression Analyses

Results from global models (OLS) and local models (GWR) are presented in Tables S2 and S3. GWR models outperformed the global OLS models (i.e., lower Akaike information criterion) and reduced spatial autocorrelation in standardized residuals. Model evaluation using the Akaike information criterion (AICc), which estimates the quality of the model, considering both the goodness of fit and complexity, showed that GWR models 2 and 3 performed better than model 1 in all cases. The AICc of model 3 was similar to model 2 for the three outcomes variables.

Considering socio-demographic characteristics, global OLS models adjusted for total energy and year of survey showed that age, gender, and nationality were significantly associated with each outcome variable. Older age and being a woman were associated with lower Na:K ratio and showed a strong positive relationship with K intake and, to a smaller extent, with Na intake. Swiss nationality was associated with higher Na:K ratio, lower Na intake, and K intake. Tertiary education was negatively associated with Na:K ratio and positively with K intake; medium-skilled occupation was negatively associated with Na intake and being married/cohabiting was associated with a slightly lower K intake. The year of survey was positively associated with K intake and negatively associated with Na intake (Table S2).

For food environment characteristics, the supermarket density was positively associated with K intake and negatively associated with Na:K ratio. However, no food environment characteristics were significantly associated with Na intake (Table S2).

Furthermore, the associations between the explanatory variable and outcome variables displayed clear spatial variability (Table S3). Several predictor variables were significant over the entire study area, while others were only significant in some areas and few were not statistically significant across the entire study area. Supermarket density was significant only in the more isolated parts of the state of Geneva (Figure S3).

### 3.3. Spatial Analyses

#### 3.3.1. Global Spatial Autocorrelation

The global spatial autocorrelation analysis using GMI statistics calculated at incremental fixed distance bands showed that the Na:K ratio exhibited the strongest spatial autocorrelation at a fixed distance band of 800 m (z-score = 1.747, *p* = 0.047). K intake showed the strongest global spatial autocorrelation at 400 m (z-score = 1.929, *p* = 0.036) (Table S4). Na intake showed no statistically significant global spatial autocorrelation at any evaluated distance and was thus evaluated at the same distance as the Na:K ratio (800 m) (Table S4).

#### 3.3.2. Local Spatial Autocorrelation

Significant local spatial clusters were detected for both unadjusted and adjusted Na:K ratio, Na, and K intakes.

**Figure 1:**
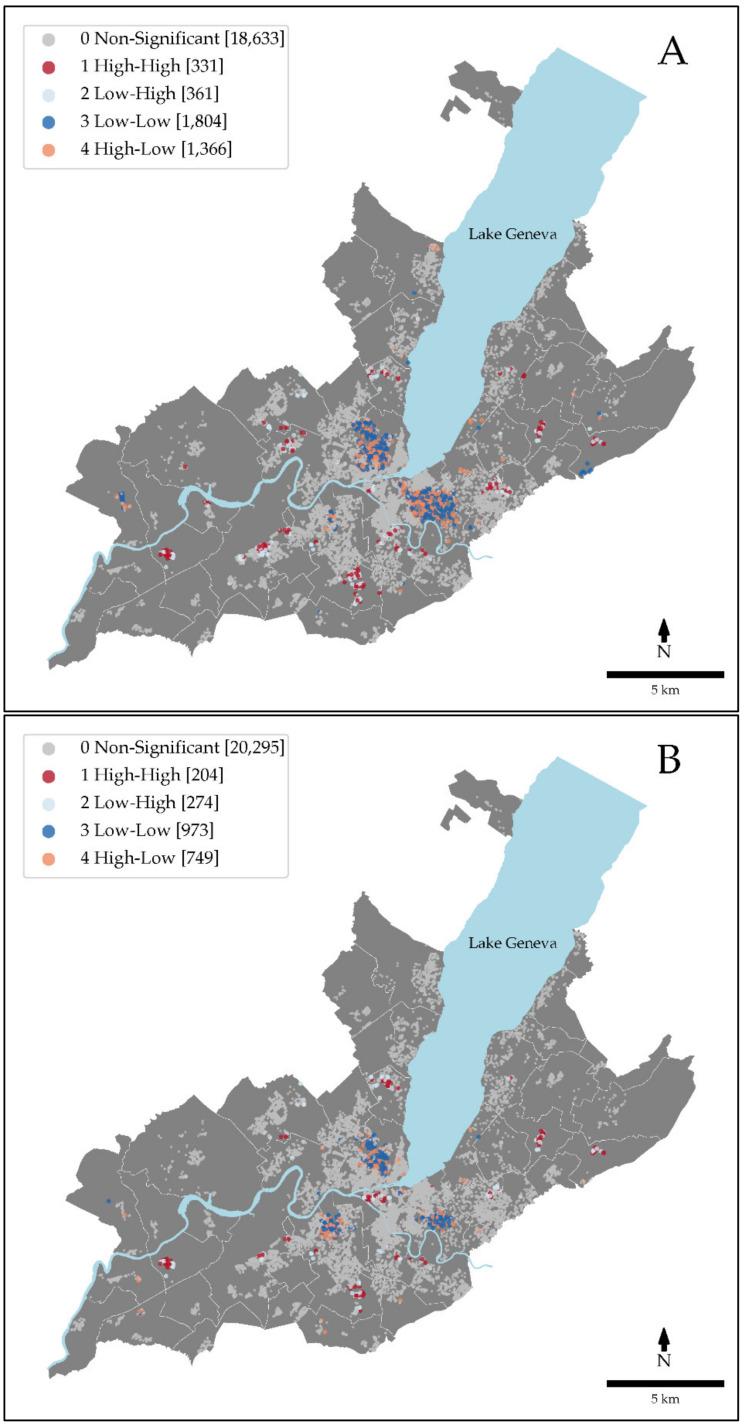
Local spatial clustering of Na:K ratio. Local Moran’s *I* spatial clusters of the Na:K ratio (**A**) unadjusted and (**B**) adjusted for socio-demographic and food environment characteristics using a geographically weighted regression (GWR model 3). The dark red markers (1 High-High) correspond to the individuals with a high Na:K ratio surrounded by individuals with a high Na:K ratio. The light blue markers (2 Low-High) correspond to individuals with a low Na:K ratio surrounded by individuals with a high Na:K ratio. The dark blue markers (3 Low-Low) correspond to individuals with a low Na:K ratio surrounded by individuals with a low Na:K ratio. The light red markers (4 High-Low) correspond to individuals with a high Na:K ratio surrounded by individuals with a low Na:K ratio. Grey markers are not significant at α = 0.05. White lines correspond to municipality delimitations.

For the unadjusted Na:K ratio, the low-low class regrouped 8% (*n* = 1,804) of the participants, mainly located in the urban parts of the state of Geneva and the high-high class grouped 1.5% (*n* = 331) of the participants, dispersed in small clusters in the rural areas (Figure 1A). After the full adjustment for both socio-demographic and food environment characteristics (GWR Model 3), spatial clusters were reduced, with 4.3% (*n* = 973) of the participants left in the low-low class and 0.9% (*n* = 204) of the participants in the high-high class (Figure 1B).

Na intake showed similar patterns, with three large low-low class clusters in urban areas grouping 5.5% (*n* = 1229) and several small high-high clusters (*n* = 203) (Figure S4A). After full adjustment (GWR Model 3), Na intake low-low class mainly disappeared, with only 1.1% (*n* = 255) of the participants remaining, while the high-high class clusters were only slightly reduced and displaced (0.9%, *n* = 196) (Figure S4B).

Finally, unadjusted K intake did not show an urban-rural divide with either low-low (4.8%, *n* = 1084) or high-high class clusters (2.1%, *n* = 481) found in urban and rural areas (Figure S5A). After full adjustment (GWR Model 3), the low-low class was thinned down to 1.9% of the participants (*n* = 433), while the high-high class almost doubled in size (3.2%, *n* = 721), with new clusters appearing and others slightly geographically displaced (Figure S5B).

Maps of the Na:K ratio, Na, and intakes adjusted for food environment characteristics (GWR Model 1) and socio-demographic characteristics (GWR Model 2) are presented in the Supplementary Materials (Figures S6–S8). Overall, adjustment for food environment characteristics (GWR Model 1) only slightly attenuated the spatial clusters, while the adjustment for socio-demographic factors (GWR Model 2) greatly influenced the spatial clustering.

#### 3.3.3. Socio-Demographic and Food Environment Differences between High-High and Low-Low Class Clusters of Unadjusted Na:K Ratio, Na, and K Intakes

**Figure 2:**
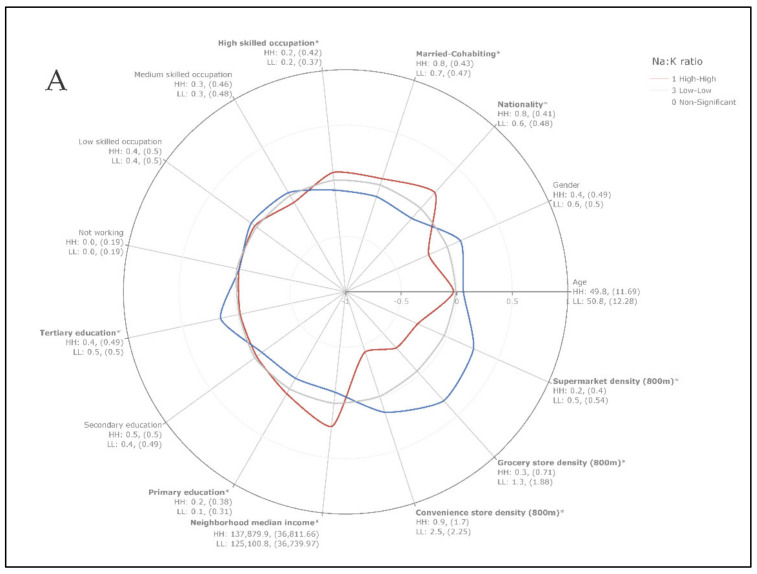

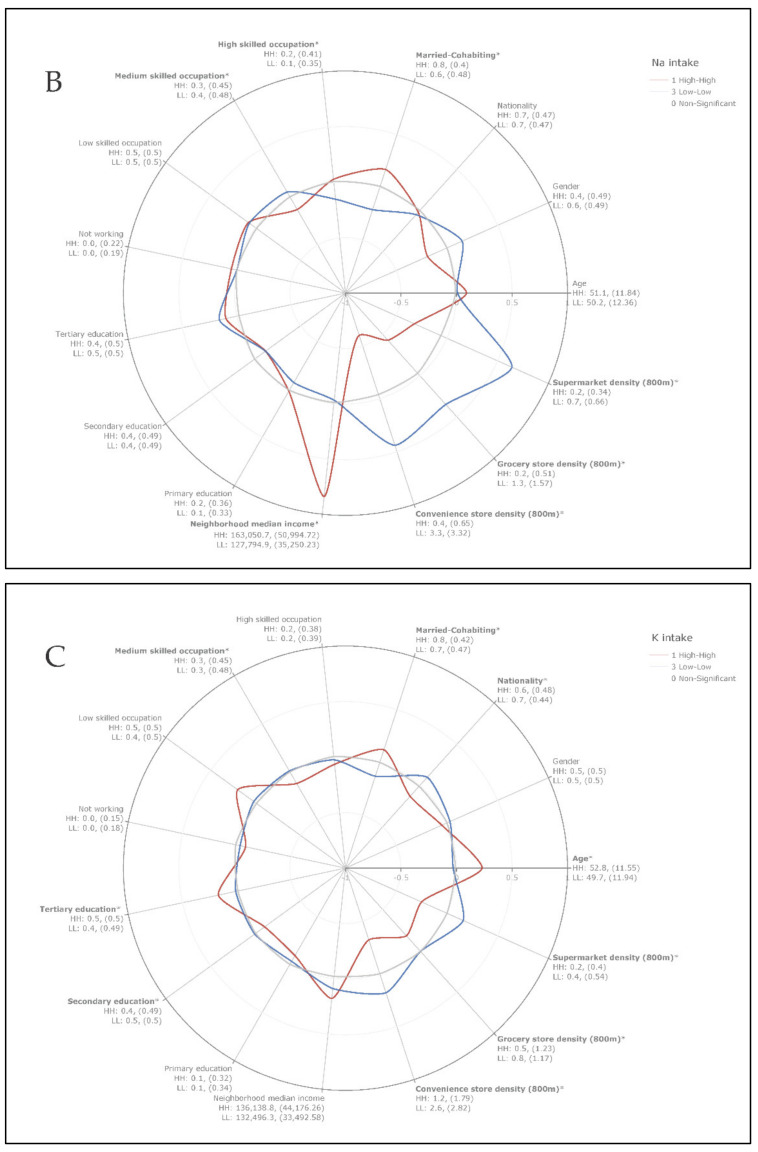
Characterization of local spatial clusters. Radar plot of standardized socio-demographic and food environment characteristics in high-high and low-low spatial clusters for (**A**) Na:K ratio, (**B**) Na intake, and (**C**) K intake. The mean (SD) is provided under each characteristic. High-high class (HH); low-low class (LL). The statistical significance was evaluated using Welch’s *t*-tests for continuous variables and Fisher’s exact tests for binary variables. * *p*-value < 0.05.

We found statistically significant differences in socio-demographic and food environment characteristics for the three outcome variables, which can be visualized with radar plots (Figure 2A–C).

For Na:K ratio clusters (Figure 2A), individuals in the high-high class had a Na:K ratio of 1.8 (SD ± 0.36), a Na intake of 4.5 g (SD ± 1.52), and a K intake of 2.5 g (SD ± 0.75 g), while individuals in the low-low class had a Na:K ratio of 1.1 (SD ± 0.24), a Na intake of 3.0 g (SD ± 1.18 g), and a K intake of 2.9 g (SD ± 1.09 g). The proportions of highly skilled occupation individuals and neighborhood median household income were higher in the high-high class. However, the proportion of individuals with a tertiary education was lower, while the proportion of individuals with a primary education was higher, suggesting a relationship with educational attainment rather than occupation level and neighborhood median household income. The proportion of married or cohabiting individuals and the proportion of individuals of Swiss nationality were significantly higher in the high-high class. Concerning food environment characteristics, individuals in the low-low class had a significantly higher walking-distance density of the three categories of food outlets, which could be explained by the urban-rural divide previously identified (Figure 1).

For Na intake clusters (Figure 2B), individuals in the high-high class had an estimated daily Na intake of 5.1 g (SD ± 1.36 g), a Na:K ratio of 1.7 (SD ± 0.47), and a K intake of 3.2 g (SD ± 1.07 g), while those in the low-low class had an estimated daily Na intake of 2.4 g (SD ± 0.65), a Na:K ratio of 1.2 (SD ± 0.39), and a K intake of 2.4 g (SD ± 0.8 g). The proportion of individuals married or cohabiting, having a high-skilled occupation, and with a neighborhood median household income was significantly higher for the high-high class. Individuals in the low-low class had a significantly higher walking-distance density of food outlets.

Finally, the comparison of differences in the spatial clusters of K intake (Figure 2C) showed that individuals in the high-high class had an estimated daily K intake of 3.6 g (SD ± 0.81), a Na:K ratio of 1.3 (SD ± 0.45), and a Na intake of 4.6 g (SD ± 1.69 g), while those in the low-low class had a daily intake of 2.1 g (SD ± 0.42), a Na:K ratio of 1.5 (SD ± 0.52), and a Na intake of 3.2 g (SD ± 1.22 g). The proportion of individuals married or cohabiting, having a tertiary education, and older age was significantly higher for the high-high class. Again, individuals in the low-low class had a significantly higher walking-distance density of food outlets.

## 4. Discussion

This study reveals statistically significant local spatial clusters of both unadjusted and adjusted Na:K ratio, Na, and K intakes in a large population-based study, representative of the state of Geneva, Switzerland (population around 500,000). In unadjusted Na:K ratio spatial clusters, Na:K ratio was slightly above the recommendation of 1.0 in the low-low class, while it was almost double the recommendation in the high-high class. Na intake was more than twice the amount recommended by the WHO guidelines of <2 g/day, while K intake was under the WHO recommendations of >3.5 g/day for individuals in the high-high class. Individuals in the low-low class of Na:K ratio, Na, and K had dietary intakes closer to the WHO recommendations, yet remained inadequate. This highlights that, despite improvements in recent decades, population-wide interventions aimed at reducing Na intake and increasing K intake remain essential [18]. Our analyses have also shown that the Na:K ratio, Na, and K intakes are associated with location-specific socio-demographic and food environment characteristics.

To the best of our knowledge, this is the first study to examine Na:K ratio, Na, and K intakes simultaneously using a fine-scale geospatial approach. Other studies have considered geographical variations of Na and K intakes but at much broader scales [20,21,22]. Spatial analysts are increasingly recognizing the importance of using small units of analysis. By using individual points, thus considering space as a continuum, we avoid the bias resulting from the modifiable areal unit problem (MAUP), which affects results when point-based measures are aggregated [54].

Our fine-scale geospatial approach adds an important level of detail by using local spatial modelling. Unlike global regression, which provides a global estimate of the relationships among explanatory and dependent variables, local models, such as GWR, account for the possible spatial variations in these relationships. In line with previous studies, local regression modelling (GWR) outperformed global models (OLS) (i.e., lower AICc and reduction of the spatial autocorrelation in the residuals) [55,56]. Recent publications have demonstrated the analytical utility of GWR for investigating a variety of health risk factors [57,58,59,60]. The results of GWR show that, even within a relatively small region (282.5 km^2^), relations between explanatory and dependent variables may have opposite directions, further highlighting the interest of a fine-scale approach.

The adjustment for socio-demographic and food environment characteristics in separate models allowed us to evaluate how these factors may explain the presence of spatial clustering. Overall, the model adjusting socio-demographic characteristics drastically modified the spatial clusters, suggesting that the identified relationships (age, gender, nationality, tertiary education, medium-skilled occupation) may explain a major part of the unadjusted spatial clusters. Inversely, the model adjusting for food environment characteristics only very slightly modified the spatial clusters, suggesting that the relationships between walking distance density (800 m) of the three categories of food outlets and the outcome variables contributed very little to the initial unadjusted spatial clusters. These findings are in agreement with previous studies that examined spatial variations in Na and K intakes in Italy and the UK [20,21,22]. These studies found that variations in Na intake were explained by a social gradient in consumption, with less advantaged groups having a significantly higher Na intake [20,21,22].

We found that higher education attainment is associated with a lower Na:K ratio. The consideration of different indicators of socioeconomic status (SES) (i.e., educational attainment, occupation, and income) allowed to precisely assess the mechanisms, linking socioeconomic exposures to dietary intakes of Na and K [61]. The identified relationship between higher education and lower Na:K ratio is in alignment with the results of a recent meta-analysis of the association between SES and Na and K intakes [62]. The median neighborhood-level household income was not significantly associated with any outcome; this could be because individual-level indicators of SES accounted for most of the variation in Na and K intakes. However, neighborhood income was significantly different between individuals in the high-high and low-low class of Na:K ratio, which could be explained by the urban-rural divide identified through the local spatial clustering analysis.

Higher supermarket density was significantly associated with lower Na:K ratio and higher K intake. Associations between diet quality and supermarket access have been previously noted in other studies [63]. The persistence of spatial clusters after adjustment for socio-demographic and food environment characteristics suggests that other explanatory variables promote the clustering of the outcome variables. For example, we only considered geographical accessibility to food outlets in the vicinity of homes and did not include measures of nonresidential accessibility or factors shaping the spatial scales of individuals (e.g., car ownership, walkability, public transports) [45]. Future studies should also consider including measures of economic accessibility, known to be associated with overall diet quality, Na, and K intakes [64,65]. Additionally, individual-level income may not have been adequately captured by using the aggregated area-level median household income.

The health benefits of reducing the Na:K ratio have been established [6]. Despite a decreasing trend in salt consumption in recent decades, the presence of a poor Na:K ratio in both high and low spatial clusters of Na:K calls for a continued effort to optimize the Na:K ratio in the general population. The reduction of the consumption of food groups found to be associated with higher Na:K could be achieved by improving food labeling [66]. Furthermore, the salt content of processed and prepared foods should be gradually reduced [19,67]. To complement these population-wide strategies, the precise identification of populations and areas presenting particularly high Na:K ratio and their detailed characterization could enable decision-makers to prioritize resource allocation and deploy improved and targeted local Na:K reduction strategies.

### Strengths and Limitations

First, we based our spatial analyses on widely used spatial statistics and the global and the local Moran’s *I*, which have been shown to be valid and robust indicators of spatial autocorrelation. Second, the evolution of the food environment and neighborhood income during the study period was considered using historical data. Third, we determined the spatial scales of our study based on objective measures of global spatial autocorrelation, measured at incremental distances. Finally, our study benefited from the use of a large population-based study, with a homogenous and dense geographical distribution across the study area.

Several limitations of the present study should be noted. First, the cross-sectional nature of our data does not permit to draw conclusions concerning causal relationships. Second, we estimated Na and K intake based on a FFQ, which is designed to rank individuals according to their Na and K intake. This could have resulted in an under- or overestimation of the actual consumption. However, we used a calibrated equation based on a validation study, with 24-h urine collection among Swiss adults to compensate for incorrect estimated Na intake. A recent study using the same FFQ and calibrated Na intake, alongside spot urine samples, saw similar intake levels [30]. Third, despite recruitment methods aimed at collecting information on a representative sample of the general population, a participation bias cannot be excluded. Still, to reduce participation bias, the Bus Santé study randomly selects residents of the canton of Geneva, offers no monetary incentives, sends multiple reminders to selected citizens, and facilitates disadvantaged populations’ participation with a mobile medical unit that covers multiple areas of the canton [27].

## 5. Conclusions

International and national programs and interventions to reduce Na intake and promote healthy food options high in K remain essential. Our findings suggest that more progress may be achieved by complementing these population-wide strategies with local place-based interventions tailored to at-risk populations.

This study provides a fine-scale identification of at-risk populations and adds to the limited knowledge on the spatial variation of the Na:K ratio, Na, and K intakes and their determinants. We identified statistically significant spatial clustering of the Na:K ratio, Na, and K intakes in a large population-based study representative of the state of Geneva, Switzerland. The GWR analyses show that spatial variations in the determinants of Na:K ratio, Na, and K intakes exist. Older age, being a woman, higher education, and having a higher access to supermarkets are associated with lower Na:K ratio, while the opposite is seen for Swiss nationality. Our findings can be used to facilitate and inform prioritized place-based interventions tailored to identified populations, which may help reduce the prevalence of hypertension-related diseases.

## Figures and Tables

**Table 1:** Individual and neighborhood characteristics of the Bus Santé study participants 1993–2018 (N = 22,495).

		Value
Age, mean (SD)		50.1 (12)
Gender, n (%)		
	Woman	11,237 (50)
	Man	11,258 (50)
Civil status, n (%)		
	Married/Cohabiting	16,192 (72)
	Not married/cohabiting	6303 (28)
Occupation, n (%)		
	Low	9530 (42)
	Medium	7587 (34)
	High	4478 (20)
	Not working	900 (4)
Education, n (%)		
	Primary	3296 (15)
	Secondary	10,252 (45)
	Tertiary	8947 (40)
Nationality, n (%)		
	Switzerland	15,624 (70)
	Other	6871 (30)
Neighborhood median household income (CHF), mean (SD)		128,831.4 (40,884.1)
Sodium and Potassium intake, mean (SD)		
	Na Intake (g/day)	3.7 (1.6)
	K Intake (g/day)	2.7 (1.0)
	Na:K Ratio	1.4 (0.5)
Food environment, mean (SD)		
	Convenience store density (800 m)	2.1 (2.8)
	Grocery store density (800 m)	0.8 (1.4)
	Supermarket density (800 m)	0.3 (0.5)
SD, standard deviation		

## Data Availability

The data presented in this study are available from the corresponding author upon reasonable request. The dataset is not publicly available due to the sensitivity of individual georeferenced data. Requests to access the dataset should be directed to Idris Guessous, Idris.Guessous@hcuge.ch.

## Supplementary Materials

The following are available online at https://www.mdpi.com/article/10.3390/nu13061798/s1. Figure S1: Density of food outlets for each Bus Santé participant calculated within a 800 m street network distance and using a linear decay function to account for decreasing attractivity as the distance increases. Categories specified using a natural breaks classification. (A) Conveniences stores, (B) grocery stores and (C) supermarkets. Figure S2: Main sources of estimated dietary Na (A) and K (B) intakes in the 1992–2018 Bus Santé (*n* = 22,495) study. Figure S3: Composite maps of significant and insignificant (grey) parameter estimates with correction for multiple dependent hypothesis tests for the explanatory variables of the geographically weighted regression model adjusting Na:K ratio for both socio-demographic and food environment characteristics (GWR Model 3). Categories specified using a natural breaks classification. (A) Intercept, (B) Total energy intake, (C) Year of survey, (D) Age, (E), Gender, (F) Nationality, (G) High skilled occupation, (H) Medium skilled occupation, (I) Low skilled occupation, (J) Tertiary education, (K) Secondary education, (L) Neighborhood median income, (M) Married-cohabiting, (N) Convenience store density (800m), (O) Grocery store density (800m) and (P) Supermarket density (800m). Figure S4: Local spatial clustering of Na intake. Local Moran’s I spatial clusters of Na intake (A) unadjusted and (B) adjusted for socio-demographic and food environment characteristics using a geographically weighted regression (GWR model 3). The dark red markers (1 High-High) correspond to the individuals with a high Na intake surrounded by individuals with a high Na intake. The light blue markers (2 Low-High) correspond to individuals with a low Na intake surrounded by individuals with a high Na intake. The dark blue markers (3 Low-Low) correspond to individuals with a low Na intake surrounded by individuals with a low Na intake. The light red markers (4 High-Low) correspond to individuals with a high Na intake surrounded by individuals with a low Na intake. Grey markers are not significant at α = 0.05. White lines correspond to municipality delimitations. Figure S5: Local spatial clustering of K intake. Local Moran’s I spatial clusters of K intake (A) unadjusted and (B) adjusted for socio-demographic and food environment characteristics using a geographically weighted regression (GWR model 3). The dark red markers (1 High-High) correspond to the individuals with a high K intake surrounded by individuals with a high K intake. The light blue markers (2 Low-High) correspond to individuals with a low K intake surrounded by individuals with a high K intake. The dark blue markers (3 Low-Low) correspond to individuals with a low K intake surrounded by individuals with a low K intake. The light red markers (4 High-Low) correspond to individuals with a high K intake surrounded by individuals with a low K intake. Grey markers are not significant at α = 0.05. White lines correspond to municipality delimitations. Figure S6: Local Moran’s I spatial clusters of Na:K ratio (A) adjusted for food environment characteristics (GWR model 1) and (B) socio-demographic characteristics (GWR model 2) using a geographically weighted regression (GWR). The dark red markers (1 High-High) correspond to the individuals with a high Na:K ratio surrounded by individuals with a high Na:K ratio. The light blue markers (2 Low-High) correspond to individuals with a low Na:K ratio surrounded by individuals with a high Na:K ratio. The dark blue markers (3 Low-Low) correspond to individuals with a low Na:K ratio surrounded by individuals with a low Na:K ratio. The light red markers (4 High-Low) correspond to individuals with a high Na:K ratio surrounded by individuals with a low Na:K ratio. Grey markers are not significant at α = 0.05. White lines correspond to municipality delimitations. Figure S7: Local Moran’s I spatial clusters of Na intake (A) adjusted for food environment characteristics (GWR model 1) and (B) socio-demographic characteristics (GWR model 2) using a geographically weighted regression (GWR). The dark red markers (1 High-High) correspond to the individuals with a high Na intake surrounded by individuals with a high Na intake. The light blue markers (2 Low-High) correspond to individuals with a low Na intake surrounded by individuals with a high Na intake. The dark blue markers (3 Low-Low) correspond to individuals with a low Na intake surrounded by individuals with a low Na intake. The light red markers (4 High-Low) correspond to individuals with a high Na intake surrounded by individuals with a low Na intake. Grey markers are not significant at α = 0.05. Black lines correspond to municipality delimitations. Figure S8: Local Moran’s I spatial clusters of K intake (A) adjusted for food environment characteristics (GWR model 1) and (B) socio-demographic characteristics (GWR model 2) using a geographically weighted regression (GWR). The dark red markers (1 High-High) correspond to the individuals with a high K intake surrounded by individuals with a high K intake. The light blue markers (2 Low-High) correspond to individuals with a low K intake surrounded by individuals with a high K intake. The dark blue markers (3 Low-Low) correspond to individuals with a low K intake surrounded by individuals with a low K intake. The light red markers (4 High-Low) correspond to individuals with a high K intake surrounded by individuals with a low K intake. White markers are not significant at α = 0.05. Black lines correspond to municipality delimitations. Table S1. Number of food outlets by period and food outlet category. The periods of participation to the Bus Santé study of each participant are matched to the closest year available in the food outlet dataset. Table S2. Global modeling (OLS) of the associations between socio-demographic and food environment characteristics, and Na:K ratio, Na and K intakes (n = 22,495), Bus santé study, Geneva, Switzerland, 1993-2018. Table S3. Local modeling (GWR) of the associations between socio-demographic and food environment characteristics, and Na:K ratio, Na and K intakes (*n* = 22,495), Bus santé study, Geneva, Switzerland, 1993-2018. Table S4. Global Moran’s I statistics, z-scores and *p*-values calculated incrementally at a fixed distance band of 200 m, 400 m, 600 m, 800 m and 1000 m.
