# Supplementary material for: Geospatial Analysis of Sodium and Potassium Intake: A Swiss Population-Based Study
Source: Nutrients. 2021 May 25;13(6):1798. doi: 10.3390/nu13061798 (PMC8229307; doi:10.3390/nu13061798)
Supplement: Supplementary file 1 [file nutrients-13-01798-s001.zip › nutrients-1187119-supplementary.pdf]

Geospatial analysis of sodium and potassium intake: a Swiss population-based study.

David De Ridder[1,2,3,4], Fabiën N. Belle[5,6], Pedro Marques-Vidal[4,7], Bélen Ponte[8], Murielle Bochud[5], Silvia Stringhini[2,4], Stéphane Joost[1,2,4,9], Idris Guessous[1,2,3,4,*]

1 Laboratory of Geographic Information Systems (LASIG), School of Architecture, Civil and Environmental Engineering (ENAC), École Polytechnique Fédérale de Lausanne (EPFL), 1015 Lausanne, Switzerland; david.deridder@unige.ch (D.D.R.); stephane.joost@epfl.ch (S.J.)
2 Unit of Population Epidemiology, Division of Primary Care Medicine, Department of Primary Care Medicine, Geneva University Hospitals, 1205 Geneva, Switzerland; Silvia.Stringhini@hcuge.ch
3 Faculty of Medicine, University of Geneva, 1205 Geneva, Switzerland
4 Group of Geographic Information Research and Analysis in Population Health (GIRAPH); Pedro-Manuel.Marques-Vidal@chuv.ch
5 Institute of Social and Preventive Medicine (ISPM), University of Bern, 3012 Bern, Switzerland; fabien.belle@ispm.unibe.ch
6 Center for Primary Care and Public Health (Unisanté), University of Lausanne, 1010 Lausanne, Switzerland; murielle.bochud@uniste.ch
7 Department of Medicine, Internal Medicine, Lausanne University Hospital, 1011 Lausanne, Switzerland
8 Service of Nephrology and Hypertension, University Hospital Geneva, 1205 Geneva, Switzerland; belen.ponte@hcuge.ch
9 La Source, School of Nursing, University of Applied Sciences and Arts Western Switzerland (HES-SO), 1004 Lausanne, Switzerland

Supplementary Material

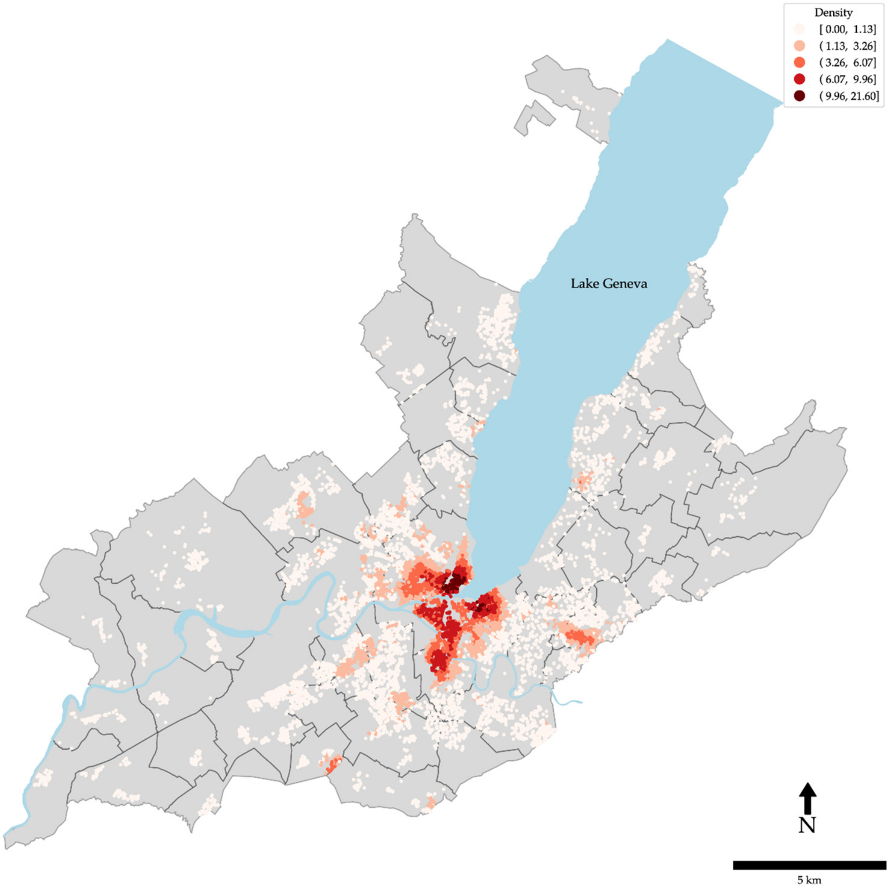

(A)

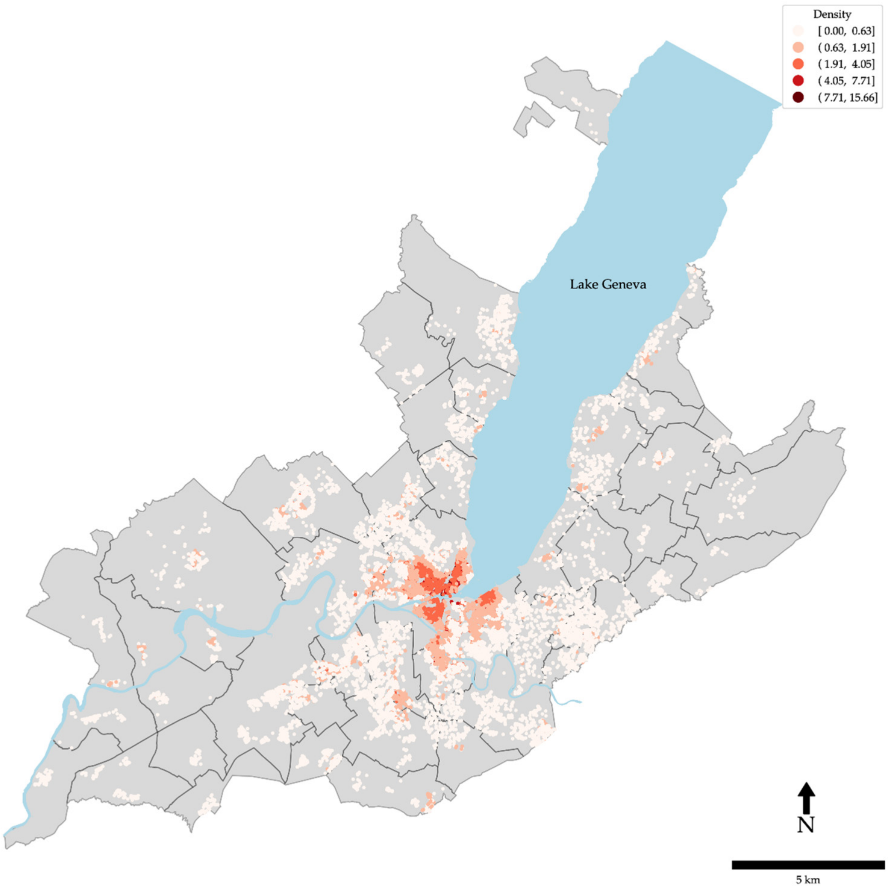
Density
[0.00, 0.63]
(0.63, 1.91]
(1.91, 4.05]
(4.05, 7.71]
(7.71, 15.66]
Lake Geneva
N
5 km

(B)

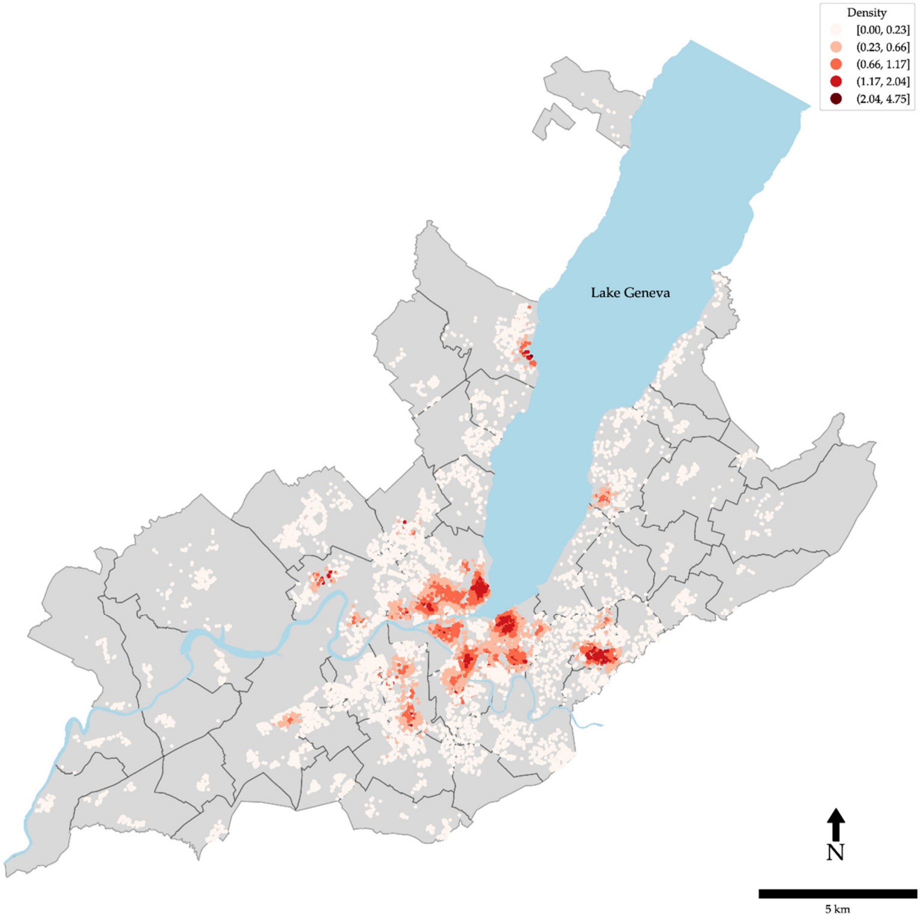

(C)

Figure S1. Density of food outlets for each Bus Santé participant calculated within a 800m street network distance and using a linear decay function to account for decreasing attractivity as the distance increases. Categories specified using a natural breaks classification. (A) Conveniences stores, (B) grocery stores and (C) supermarkets.

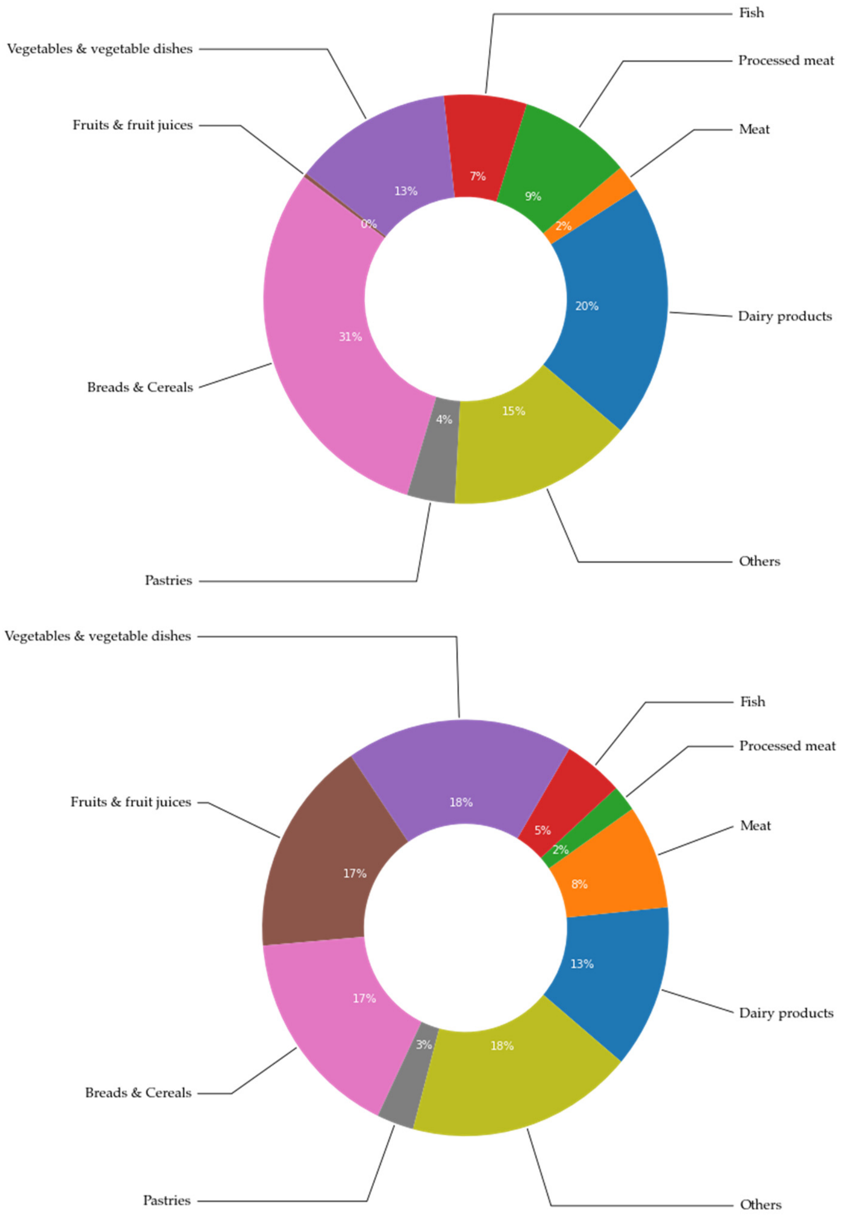

Figure S2 – Main sources of estimated dietary Na (A) and K (B) intakes in the 1992-2018 Bus Santé (n = 22,495) study.

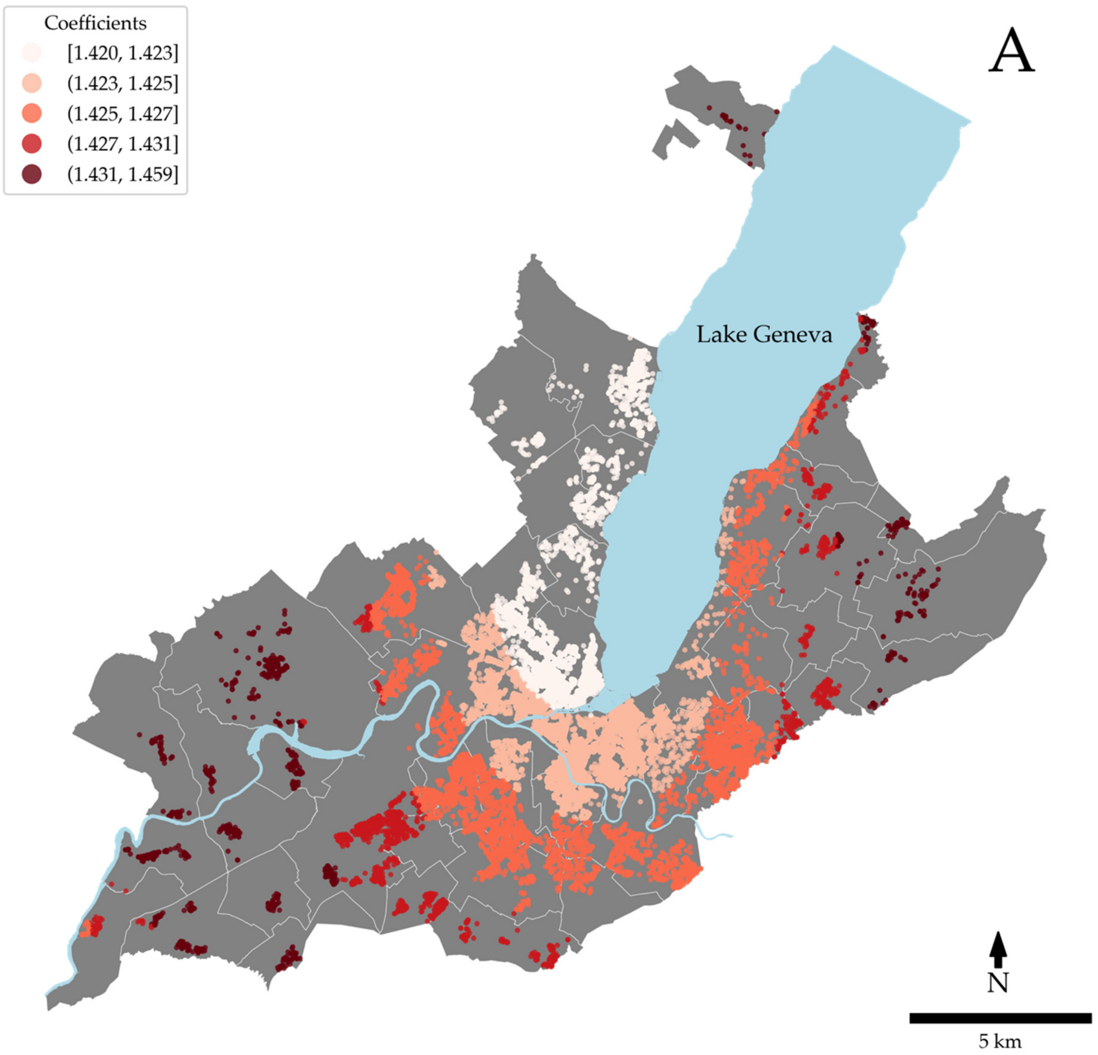
Coefficients
[1.420, 1.423]
(1.423, 1.425]
(1.425, 1.427]
(1.427, 1.431]
(1.431, 1.459]
A
Lake Geneva
N
5 km

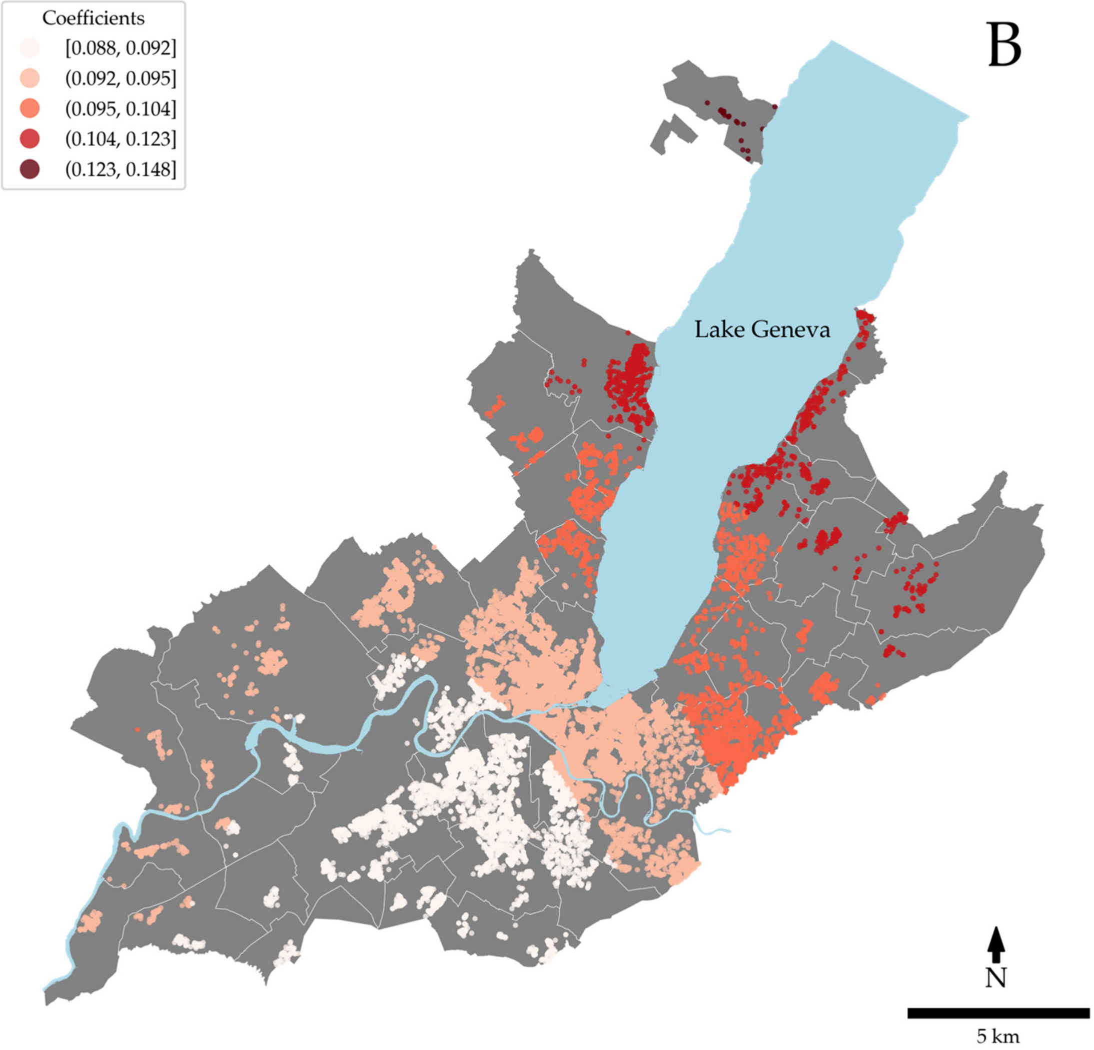
Coefficients
[0.088, 0.092]
(0.092, 0.095]
(0.095, 0.104]
(0.104, 0.123]
(0.123, 0.148]
B
Lake Geneva
N
5 km

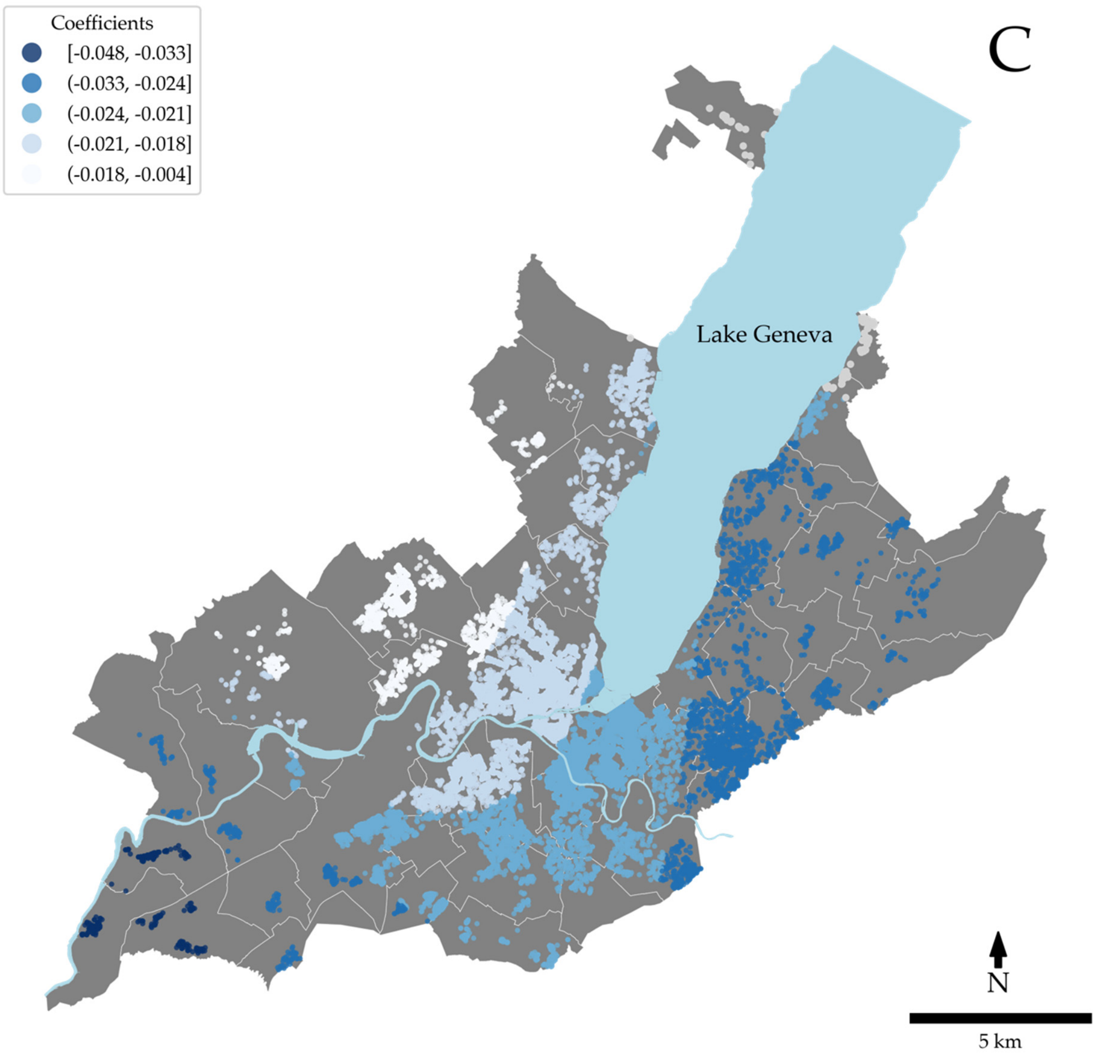
Coefficients
[-0.048, -0.033]
(-0.033, -0.024]
(-0.024, -0.021]
(-0.021, -0.018]
(-0.018, -0.004]
C
Lake Geneva
N
5 km

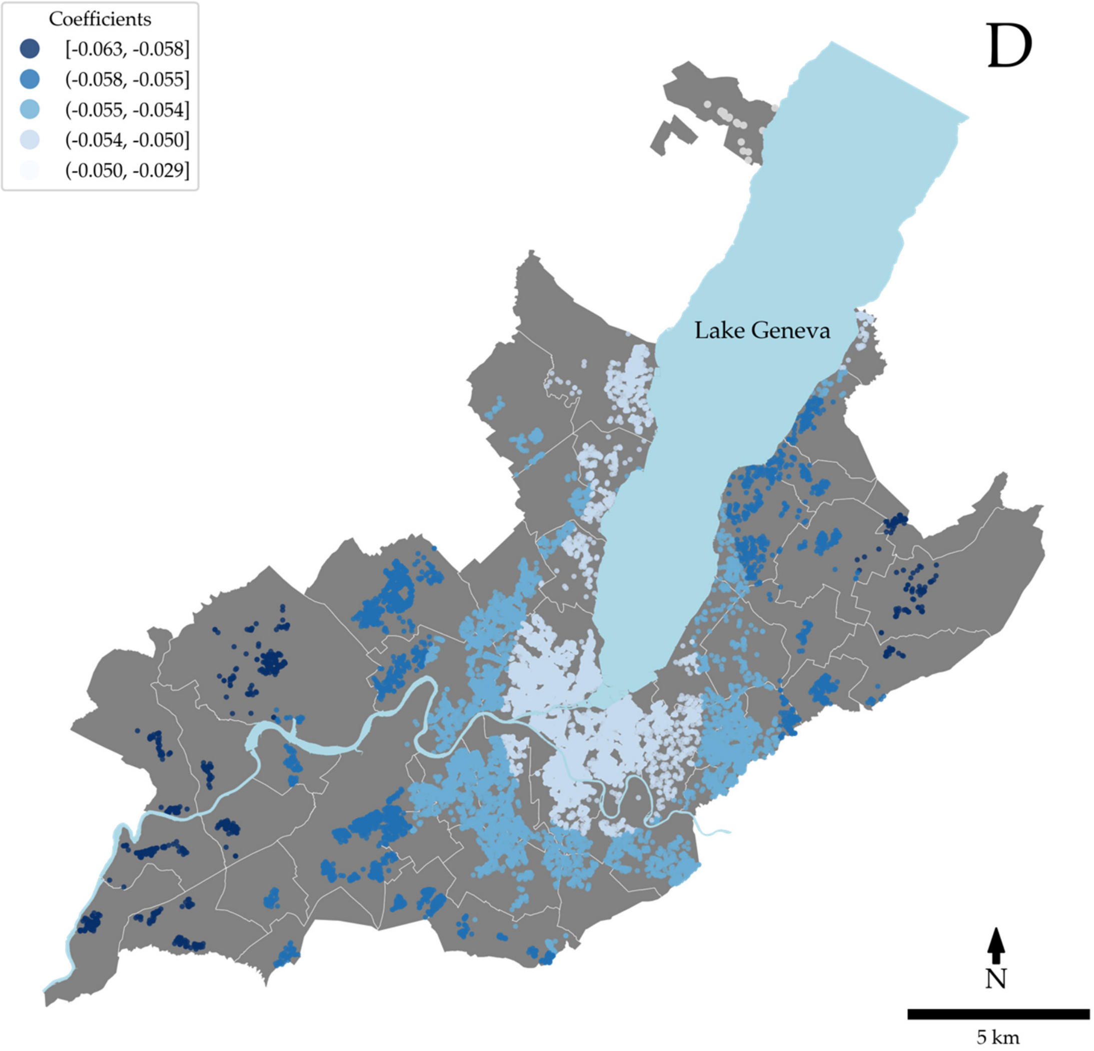
Coefficients
[-0.063, -0.058]
(-0.058, -0.055]
(-0.055, -0.054]
(-0.054, -0.050]
(-0.050, -0.029]
D
Lake Geneva
N
5 km

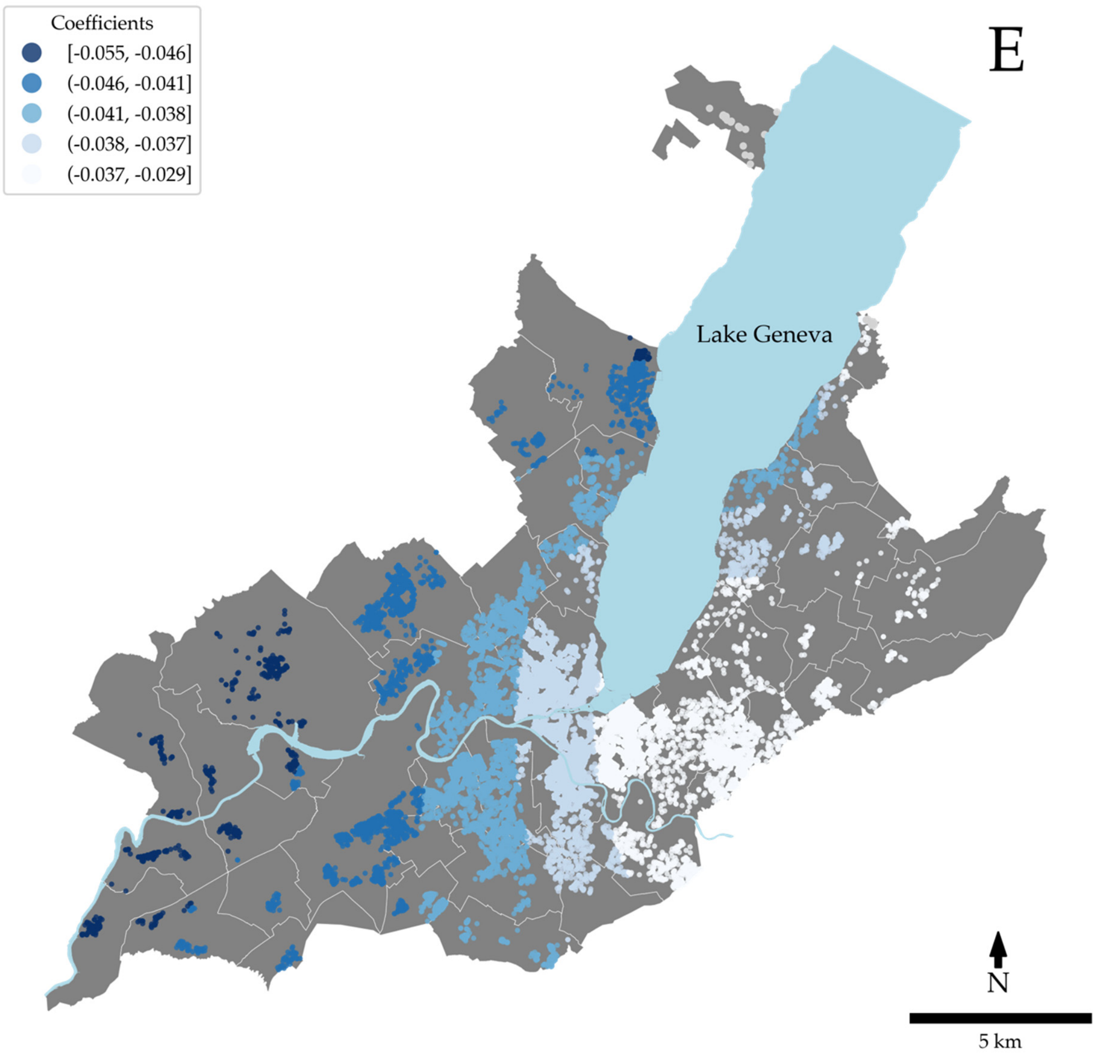
Coefficients
[-0.055, -0.046]
(-0.046, -0.041]
(-0.041, -0.038]
(-0.038, -0.037]
(-0.037, -0.029]
E
Lake Geneva
N
5 km

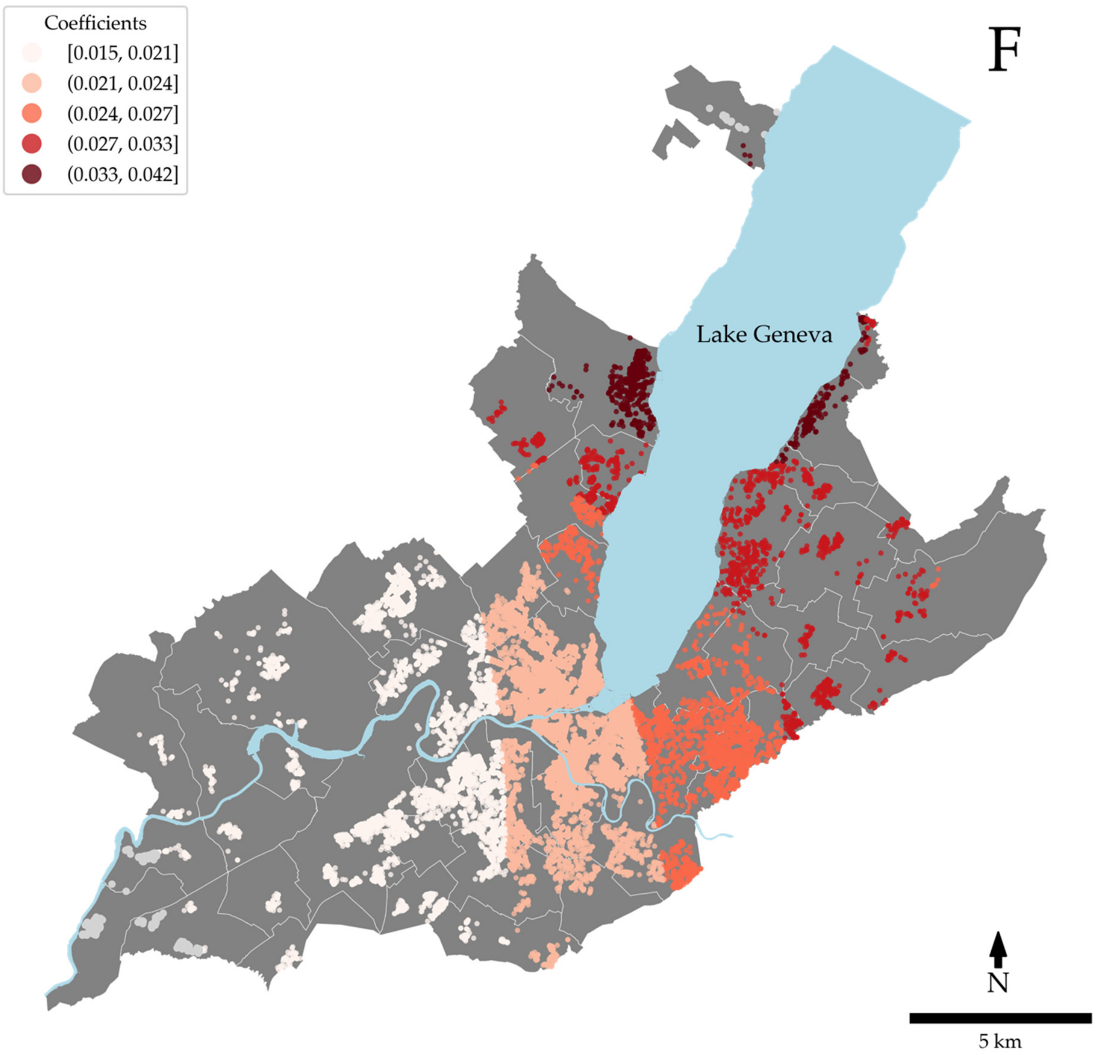
Coefficients
[0.015, 0.021]
(0.021, 0.024]
(0.024, 0.027]
(0.027, 0.033]
(0.033, 0.042]
F
Lake Geneva
N
5 km

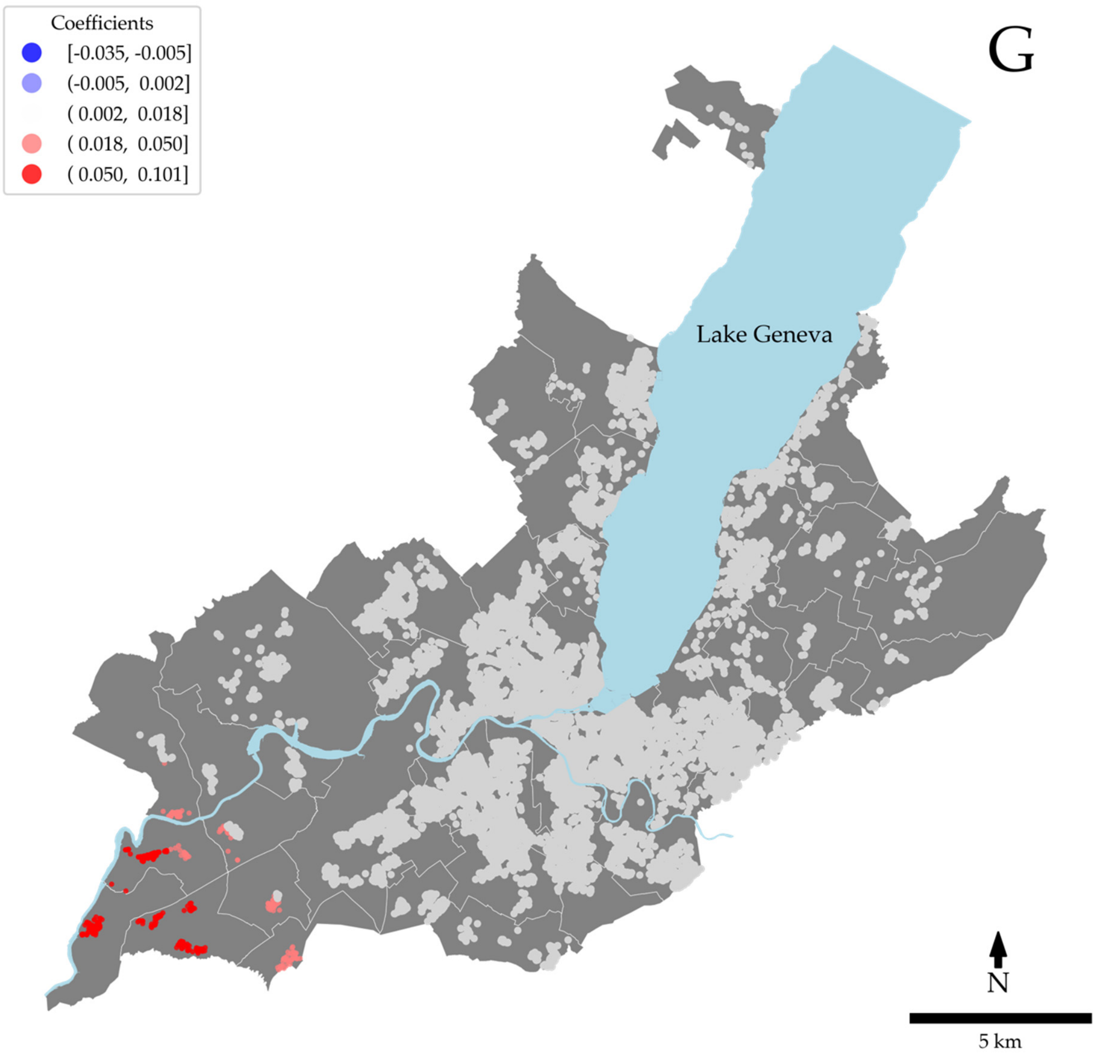

Coefficients
[-0.035, -0.005]
(-0.005, 0.002]
(0.002, 0.018]
(0.018, 0.050]
(0.050, 0.101]
G
Lake Geneva
N
5 km

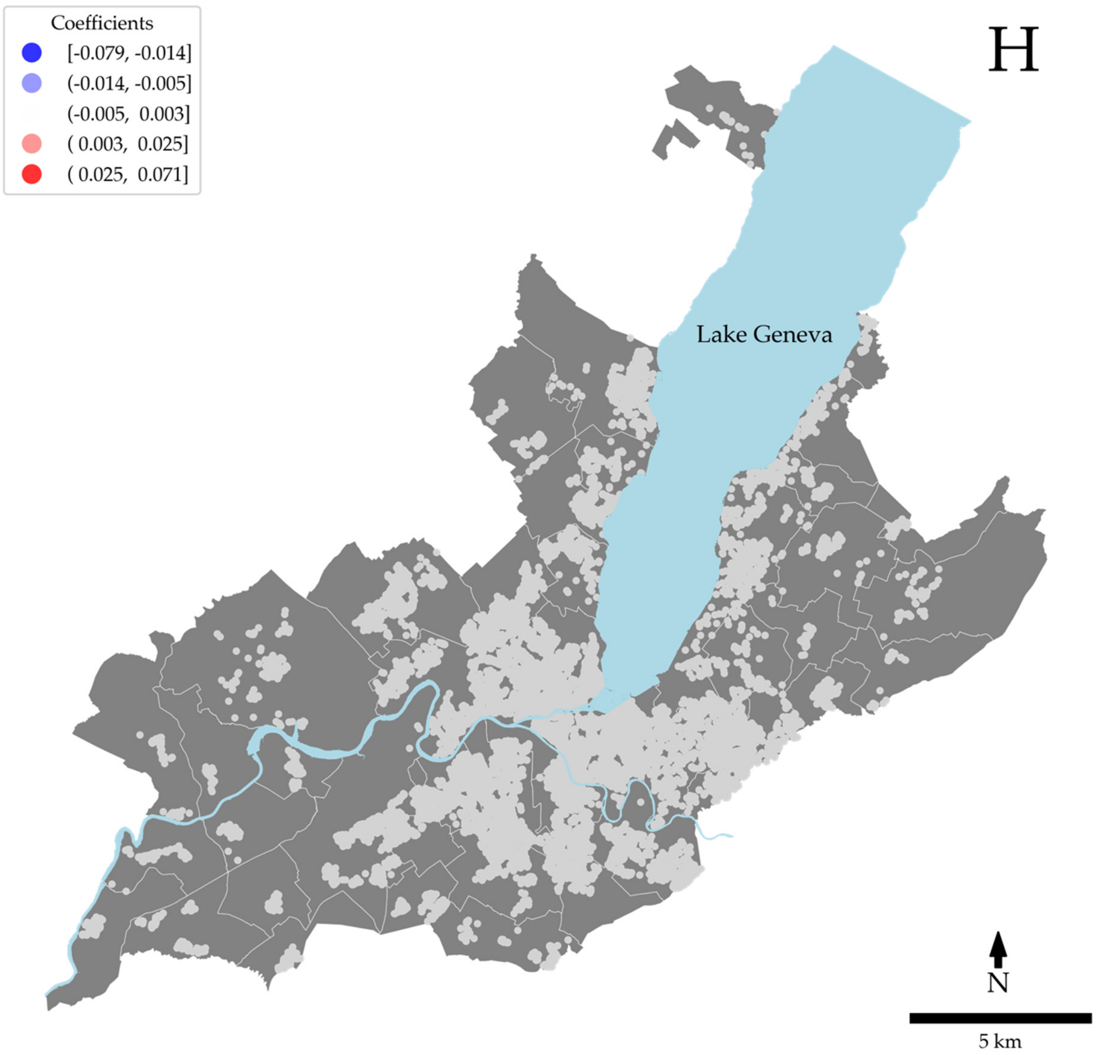

Coefficients
[-0.079, -0.014]
(-0.014, -0.005]
(-0.005, 0.003]
(0.003, 0.025]
(0.025, 0.071]
H
Lake Geneva
N
5 km

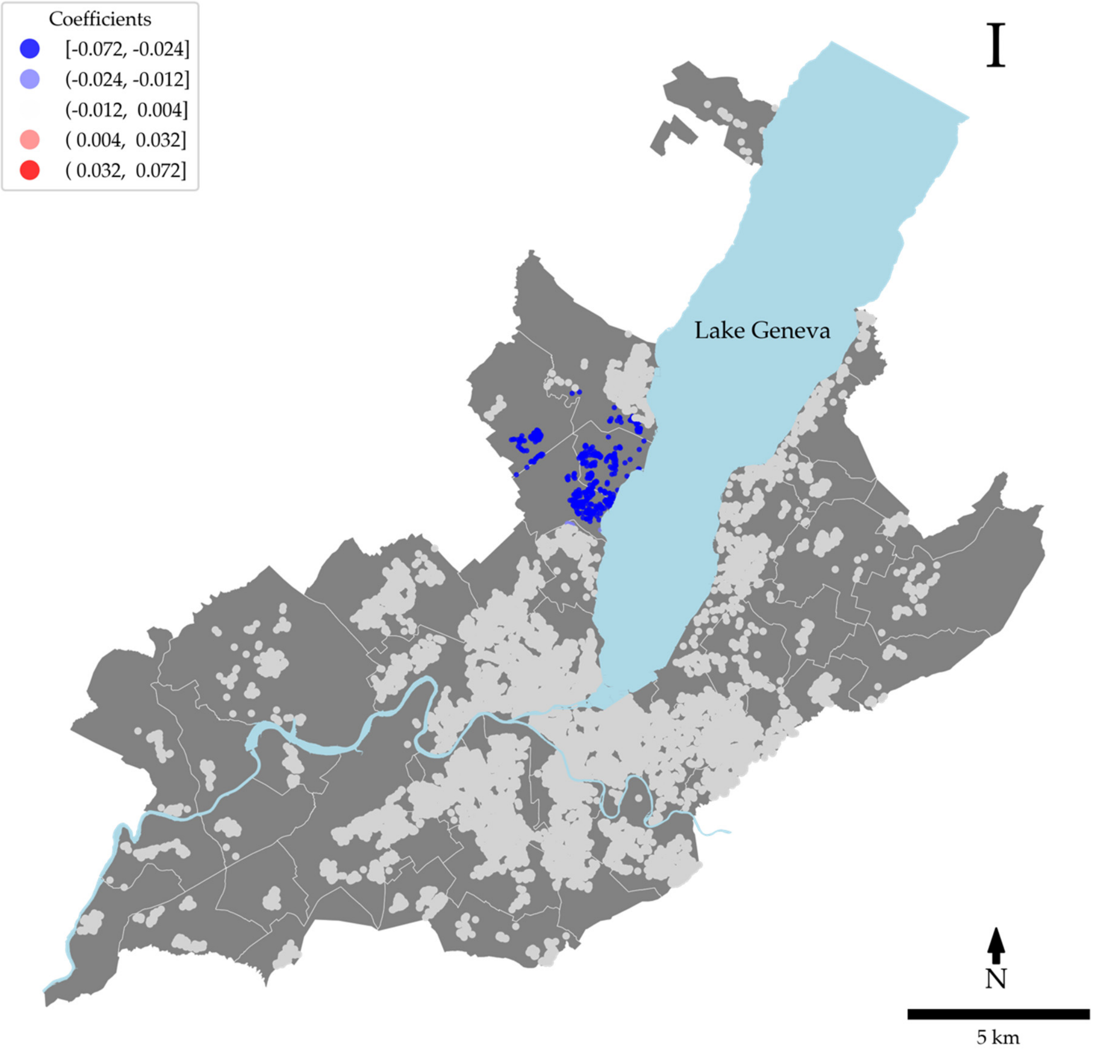
Coefficients
[-0.072, -0.024]
(-0.024, -0.012]
(-0.012, 0.004]
(0.004, 0.032]
(0.032, 0.072]
I
Lake Geneva
N
5 km

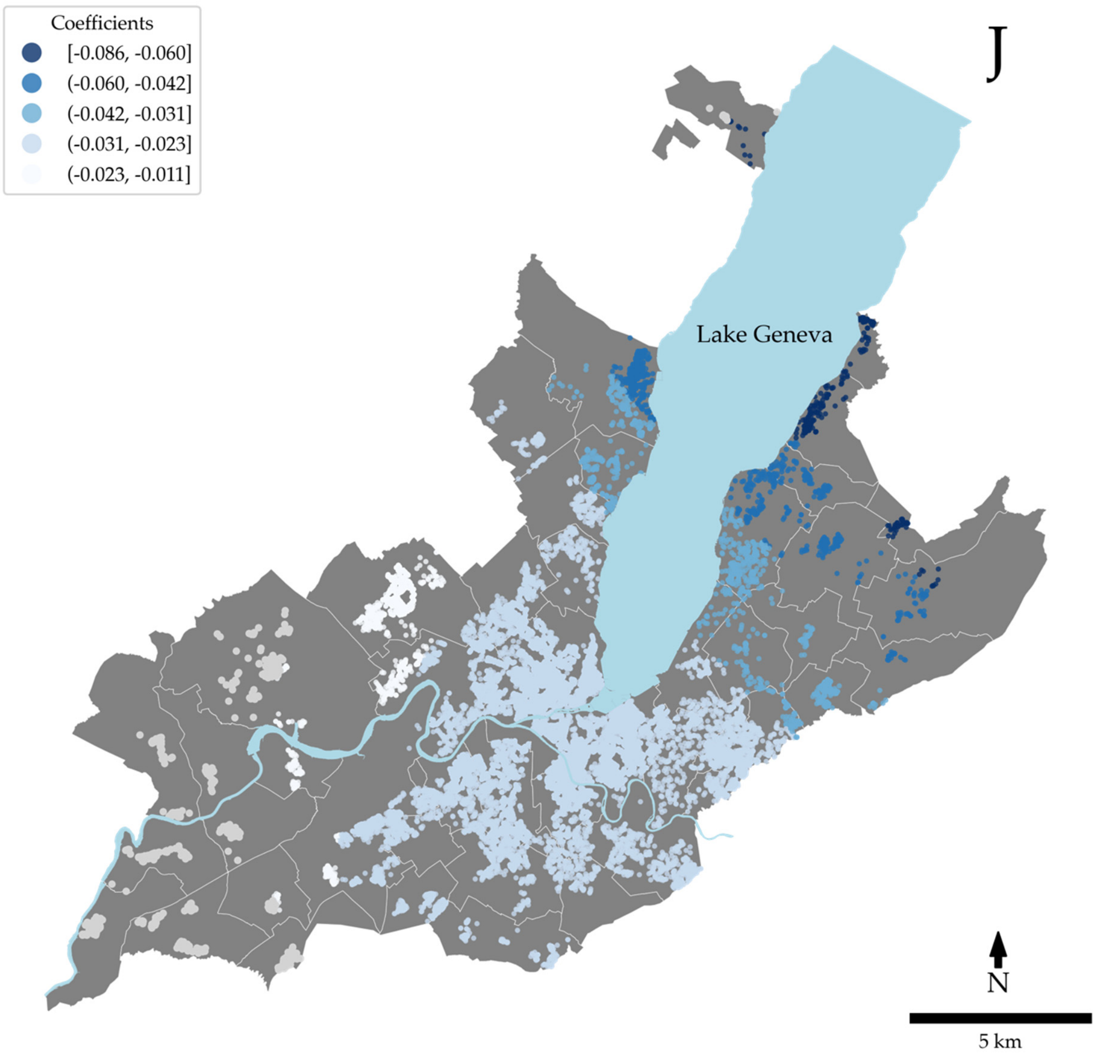
Coefficients
[-0.086, -0.060]
(-0.060, -0.042]
(-0.042, -0.031]
(-0.031, -0.023]
(-0.023, -0.011]
J
Lake Geneva
N
5 km

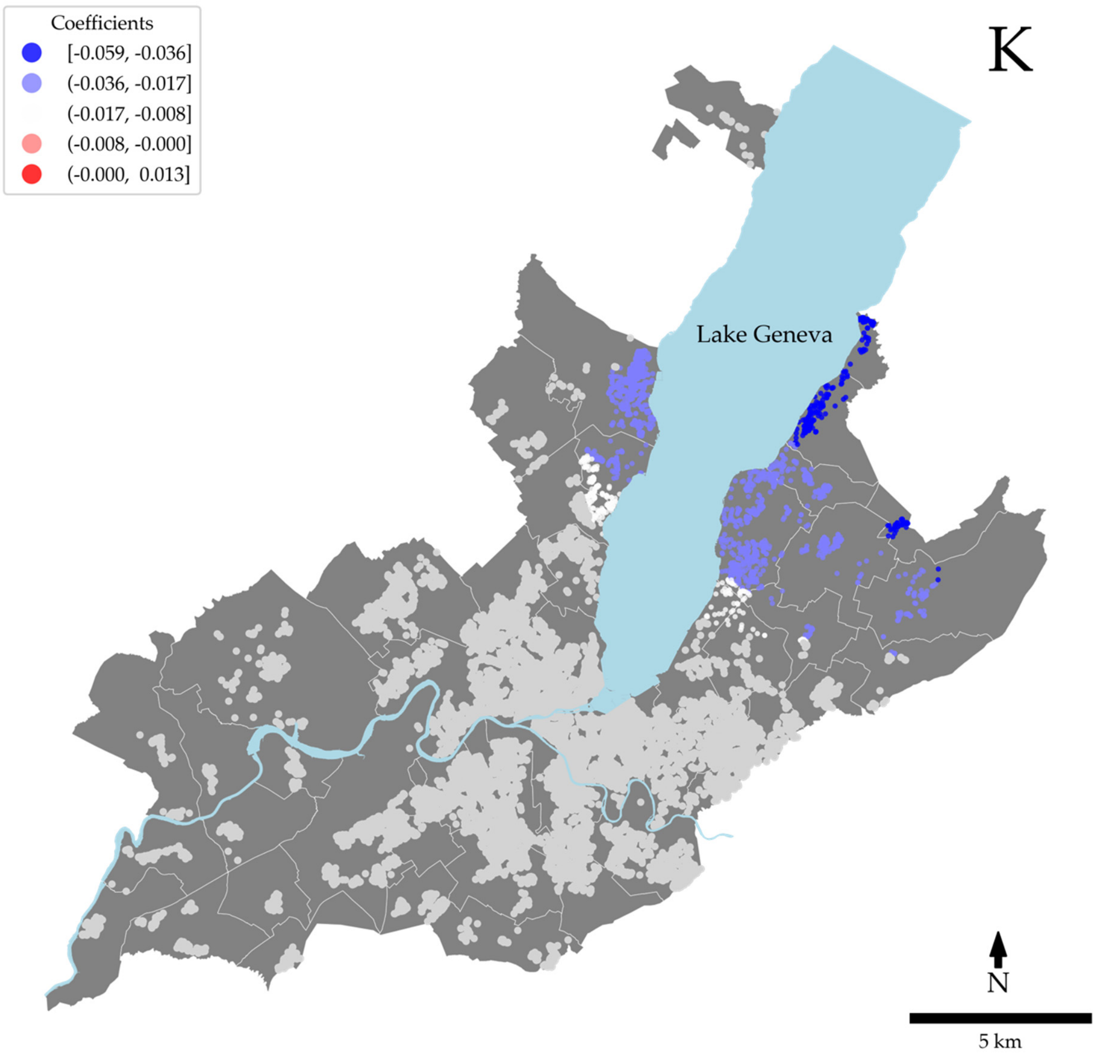
Coefficients
[-0.059, -0.036]
(-0.036, -0.017]
(-0.017, -0.008]
(-0.008, -0.000]
(-0.000, 0.013]
K
Lake Geneva
N
5 km

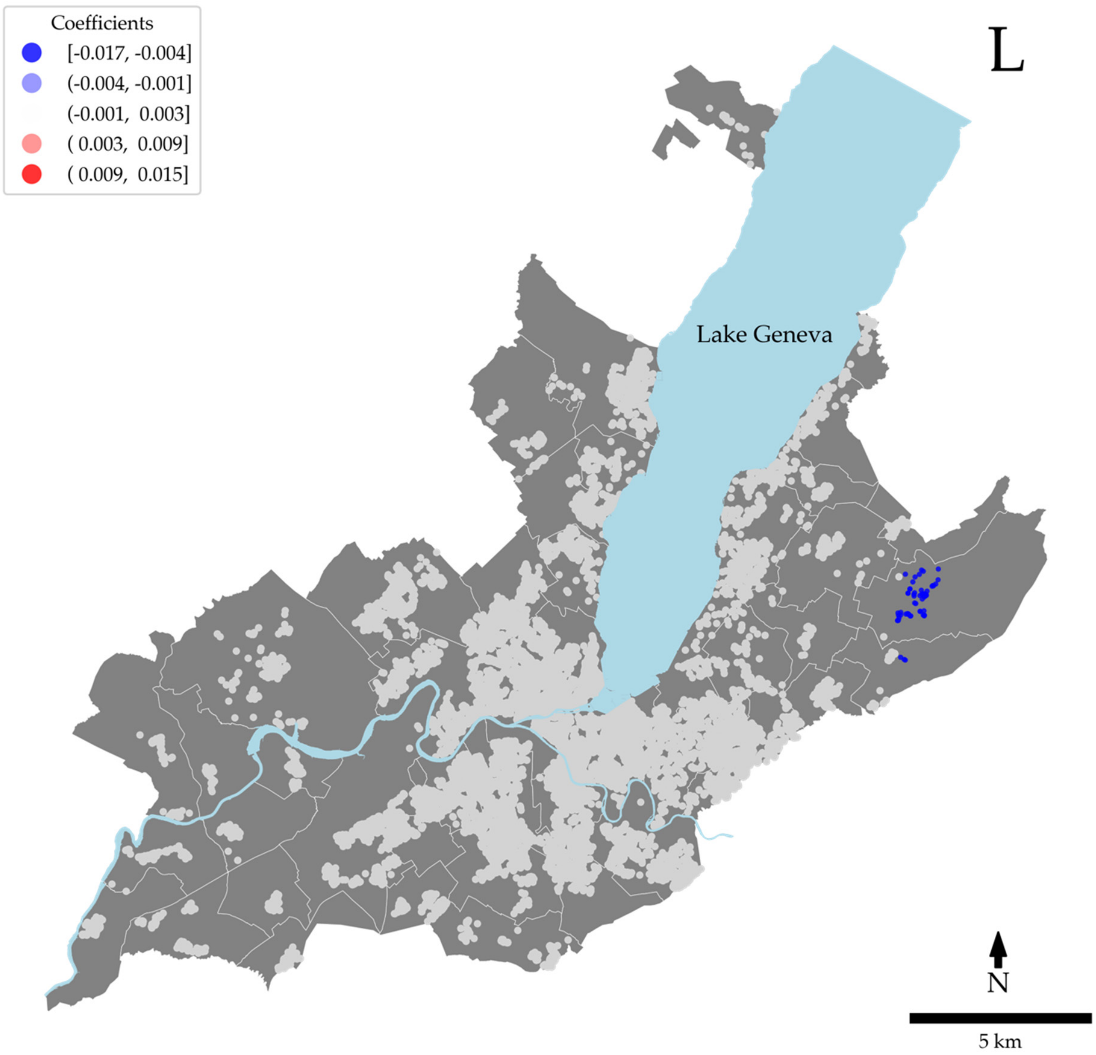
Coefficients
[-0.017, -0.004]
(-0.004, -0.001]
(-0.001, 0.003]
(0.003, 0.009]
(0.009, 0.015]
L
Lake Geneva
N
5 km

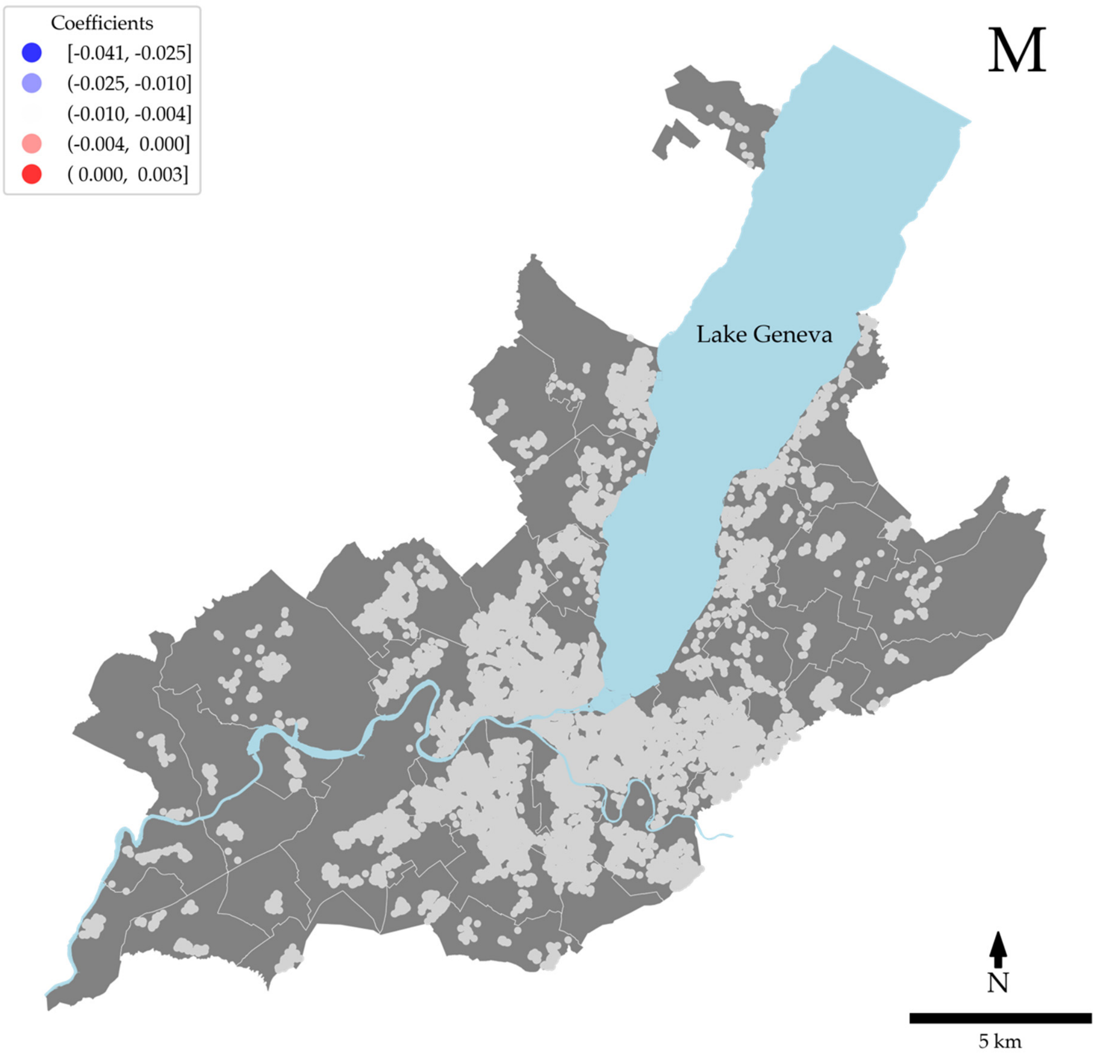
Coefficients
[-0.041, -0.025]
(-0.025, -0.010]
(-0.010, -0.004]
(-0.004, 0.000]
(0.000, 0.003]
M
Lake Geneva
N
5 km

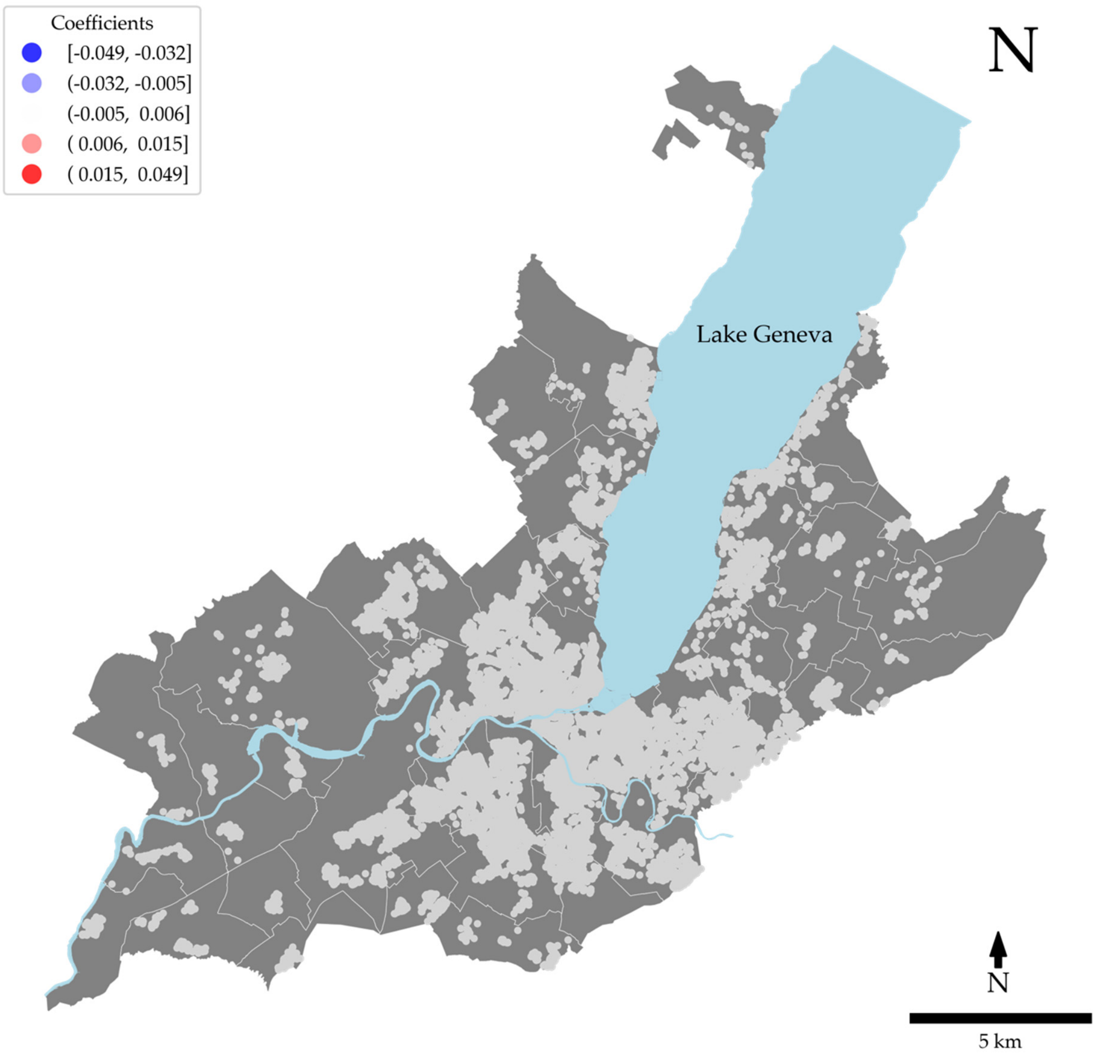
Coefficients
[-0.049, -0.032]
(-0.032, -0.005]
(-0.005, 0.006]
(0.006, 0.015]
(0.015, 0.049]
N
Lake Geneva
N
5 km

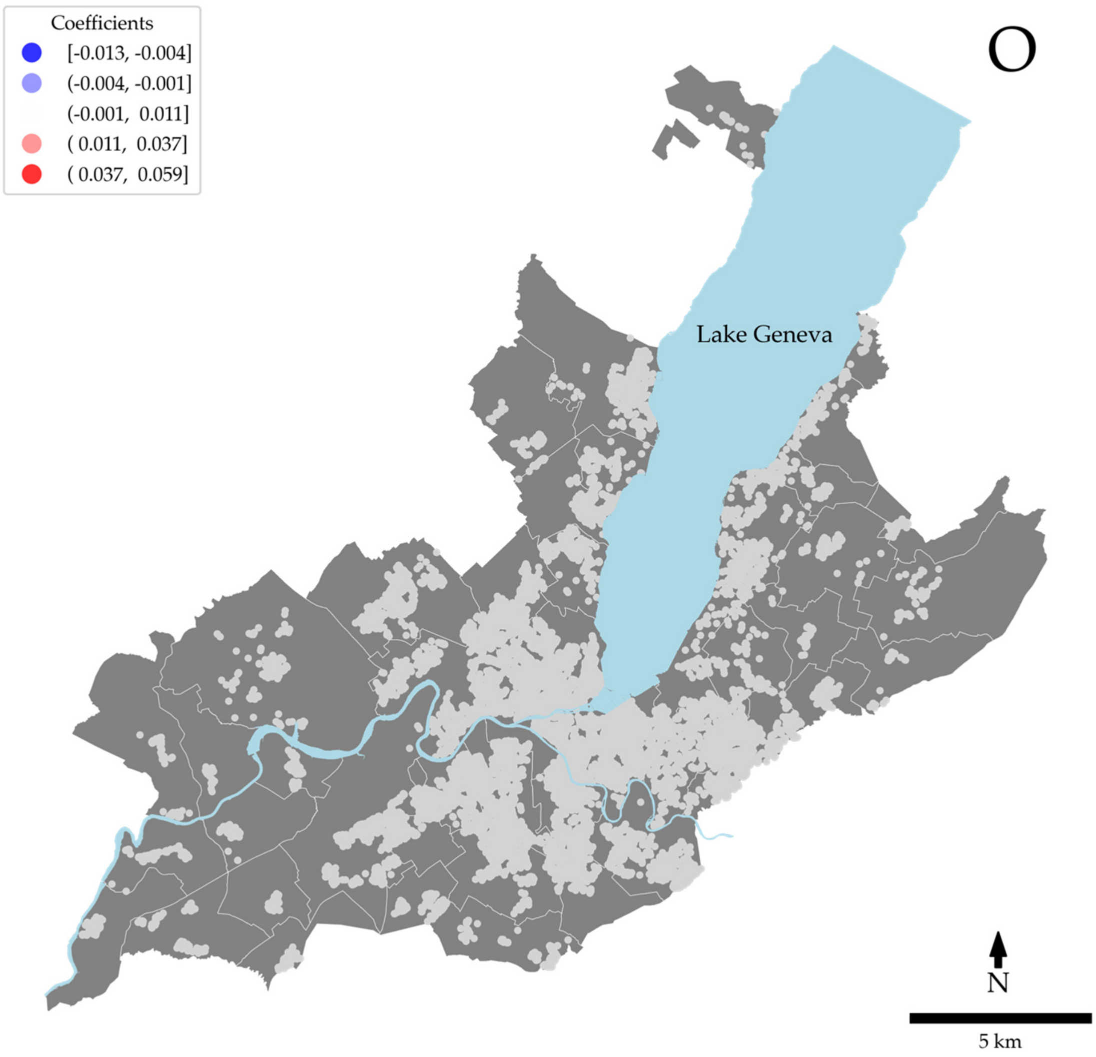
Coefficients
[-0.013, -0.004]
(-0.004, -0.001]
(-0.001, 0.011]
(0.011, 0.037]
(0.037, 0.059]
O
Lake Geneva
N
5 km

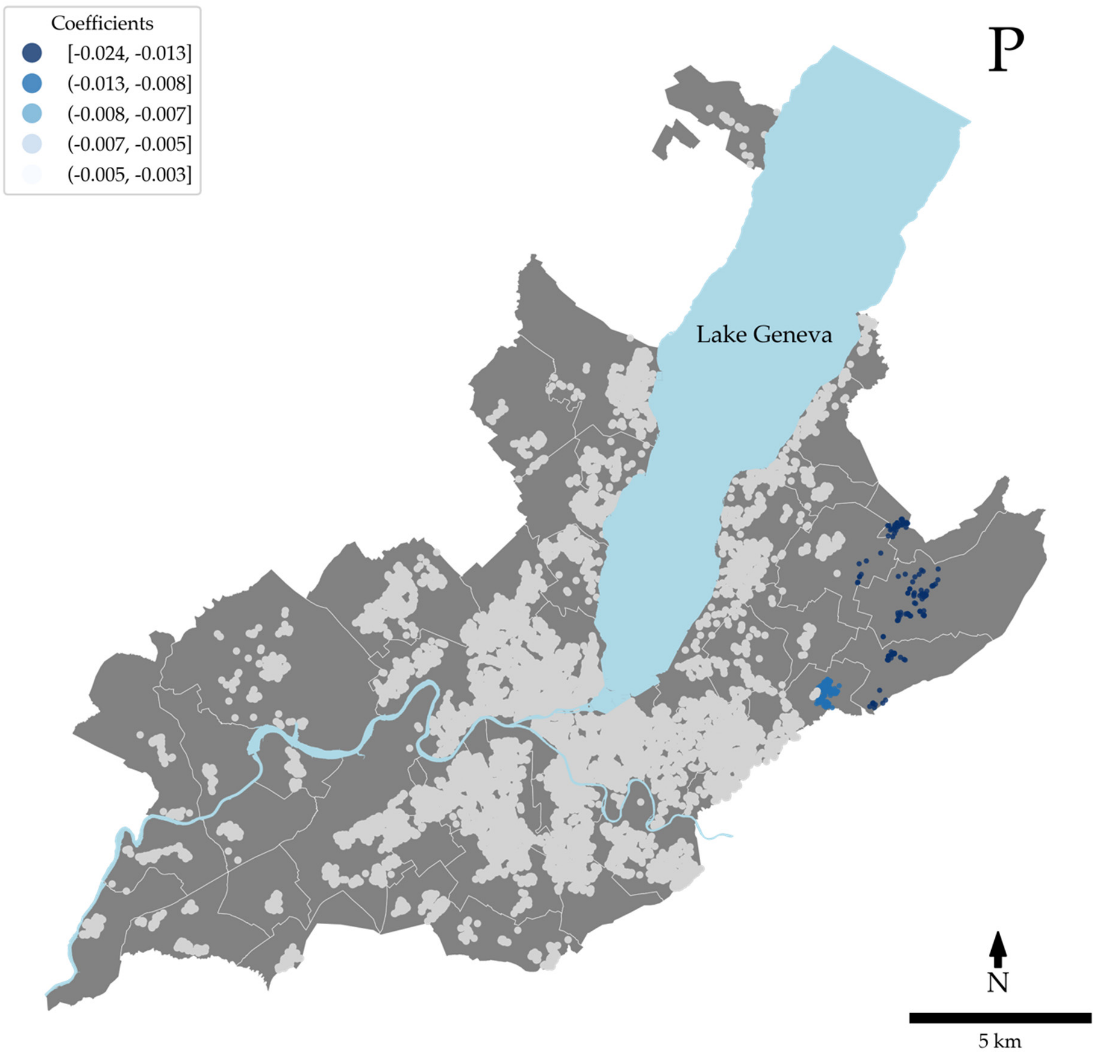

Figure S3. Composite maps of significant and insignificant (grey) parameter estimates with correction for multiple dependent hypothesis tests for the explanatory variables of the geographically weighted regression model adjusting Na:K ratio for both socio-demographic and food environment characteristics (GWR Model 3). Categories specified using a natural breaks classification. (A) Intercept, (B) Total energy intake, (C) Year of survey, (D) Age, (E), Gender, (F) Nationality, (G) High skilled occupation, (H) Medium skilled occupation, (I) Low skilled occupation, (J) Tertiary education, (K) Secondary education, (L) Neighborhood median income, (M) Married-cohabiting, (N) Convenience store density (800m), (O) Grocery store density (800m) and (P) Supermarket density (800m).

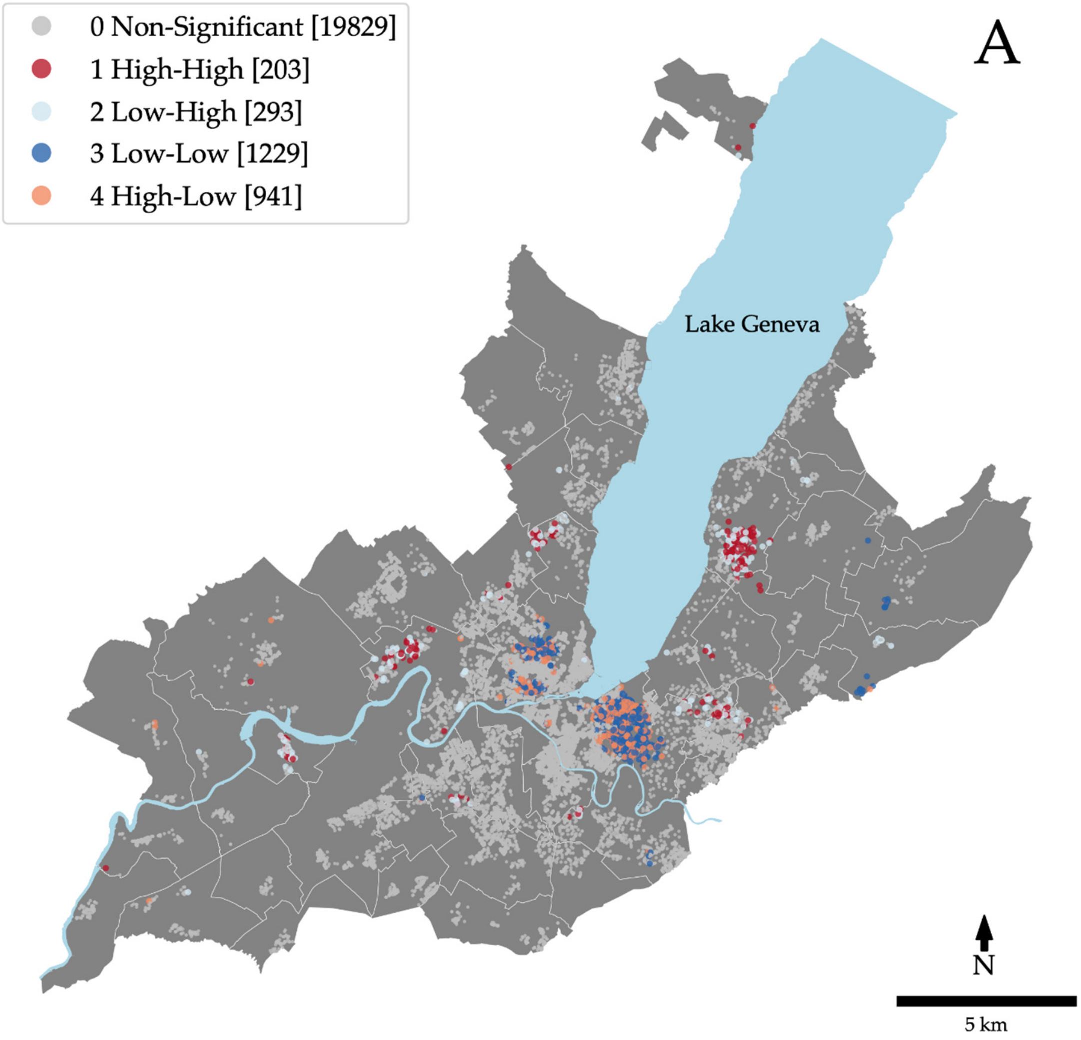
0 Non-Significant [19829]
1 High-High [203]
2 Low-High [293]
3 Low-Low [1229]
4 High-Low [941]
A
Lake Geneva
N
5 km

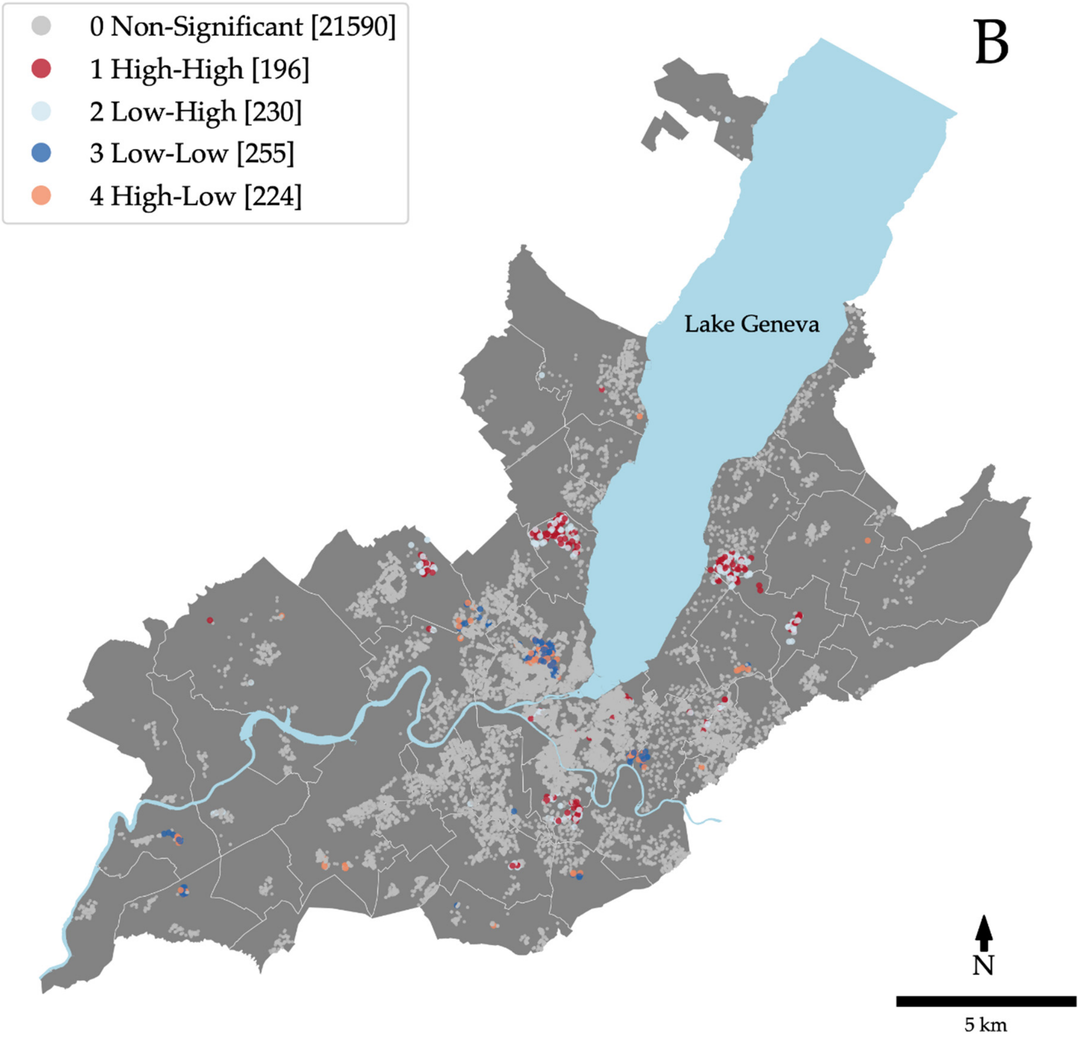

Figure S4. Local spatial clustering of Na intake. Local Moran's I spatial clusters of Na intake (A) unadjusted and (B) adjusted for socio-demographic and food environment characteristics using a geographically weighted regression (GWR model 3). The dark red markers (1 High-High) correspond to the individuals with a high Na intake surrounded by individuals with a high Na intake. The light blue markers (2 Low-High) correspond to individuals with a low Na intake surrounded by individuals with a high Na intake. The dark blue markers (3 Low-Low) correspond to individuals with a low Na intake surrounded by individuals with a low Na intake. The light red markers (4 High-Low) correspond to individuals with a high Na intake surrounded by individuals with a low Na intake. Grey markers are not significant at $\alpha = 0.05$. White lines correspond to municipality delimitations.

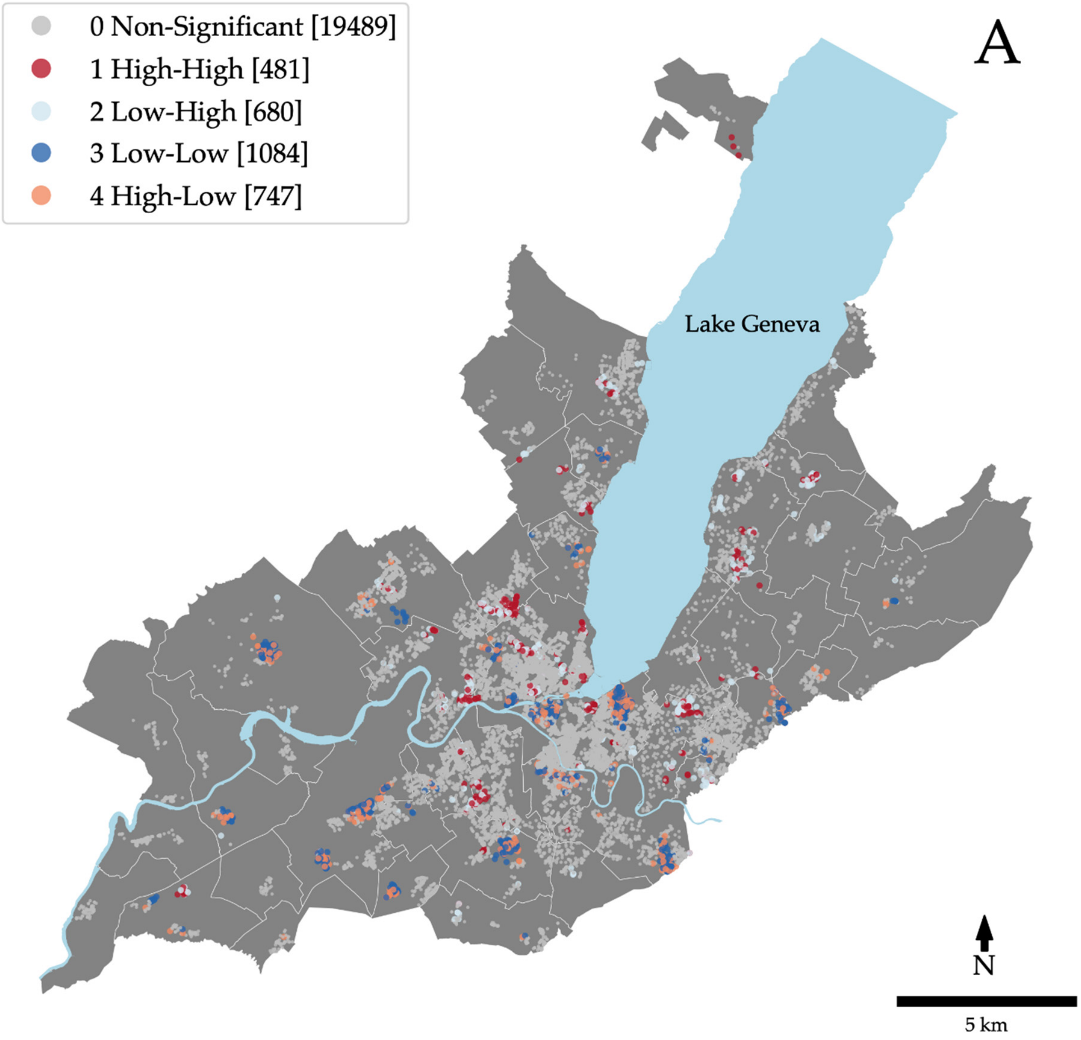
0 Non-Significant [19489]
1 High-High [481]
2 Low-High [680]
3 Low-Low [1084]
4 High-Low [747]
A
Lake Geneva
N
5 km

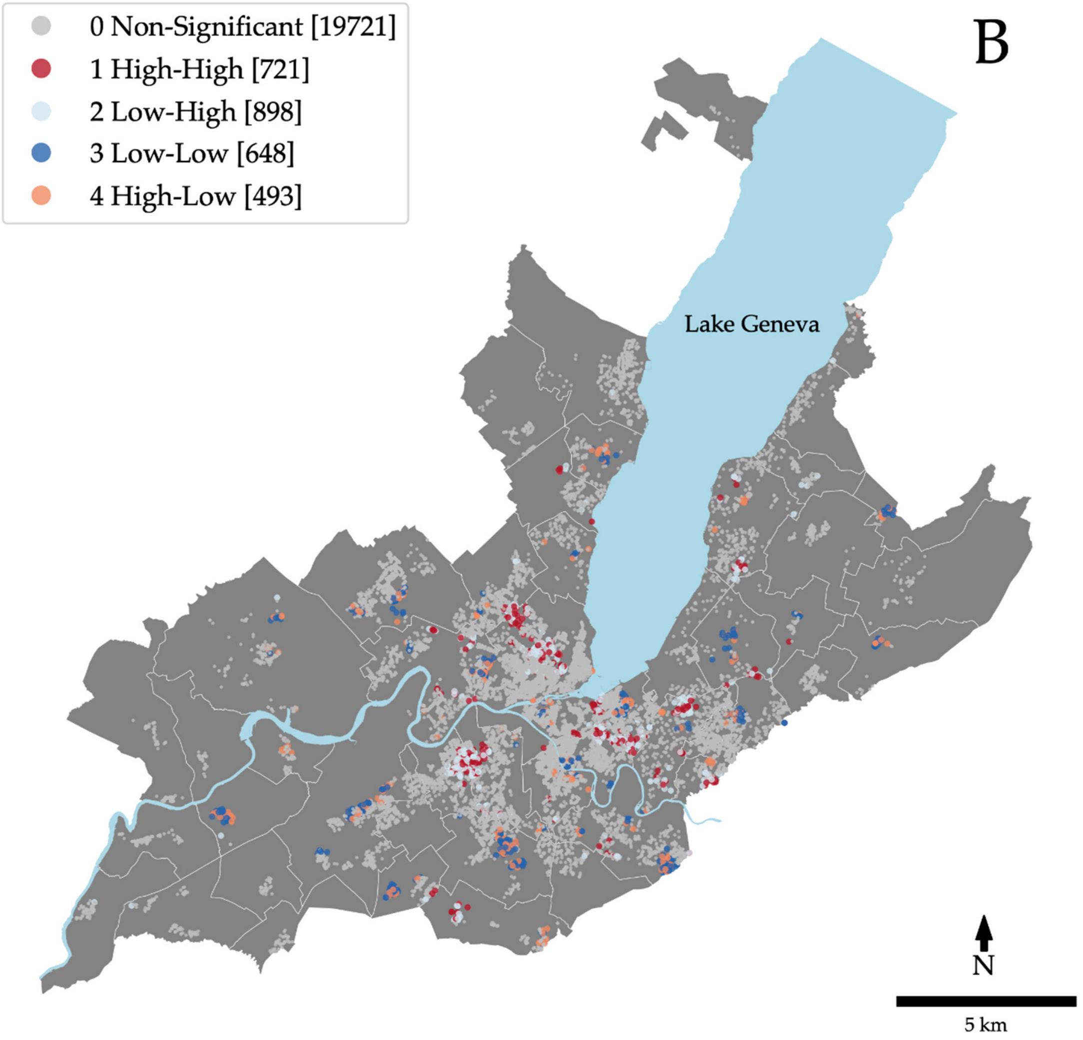

Figure S5. Local spatial clustering of K intake. Local Moran's I spatial clusters of K intake (A) unadjusted and (B) adjusted for socio-demographic and food environment characteristics using a geographically weighted regression (GWR model 3). The dark red markers (1 High-High) correspond to the individuals with a high K intake surrounded by individuals with a high K intake. The light blue markers (2 Low-High) correspond to individuals with a low K intake surrounded by individuals with a high K intake. The dark blue markers (3 Low-Low) correspond to individuals with a low K intake surrounded by individuals with a low K intake. The light red markers (4 High-Low) correspond to individuals with a high K intake surrounded by individuals with a low K intake. Grey markers are not significant at $\alpha = 0.05$. White lines correspond to municipality delimitations.

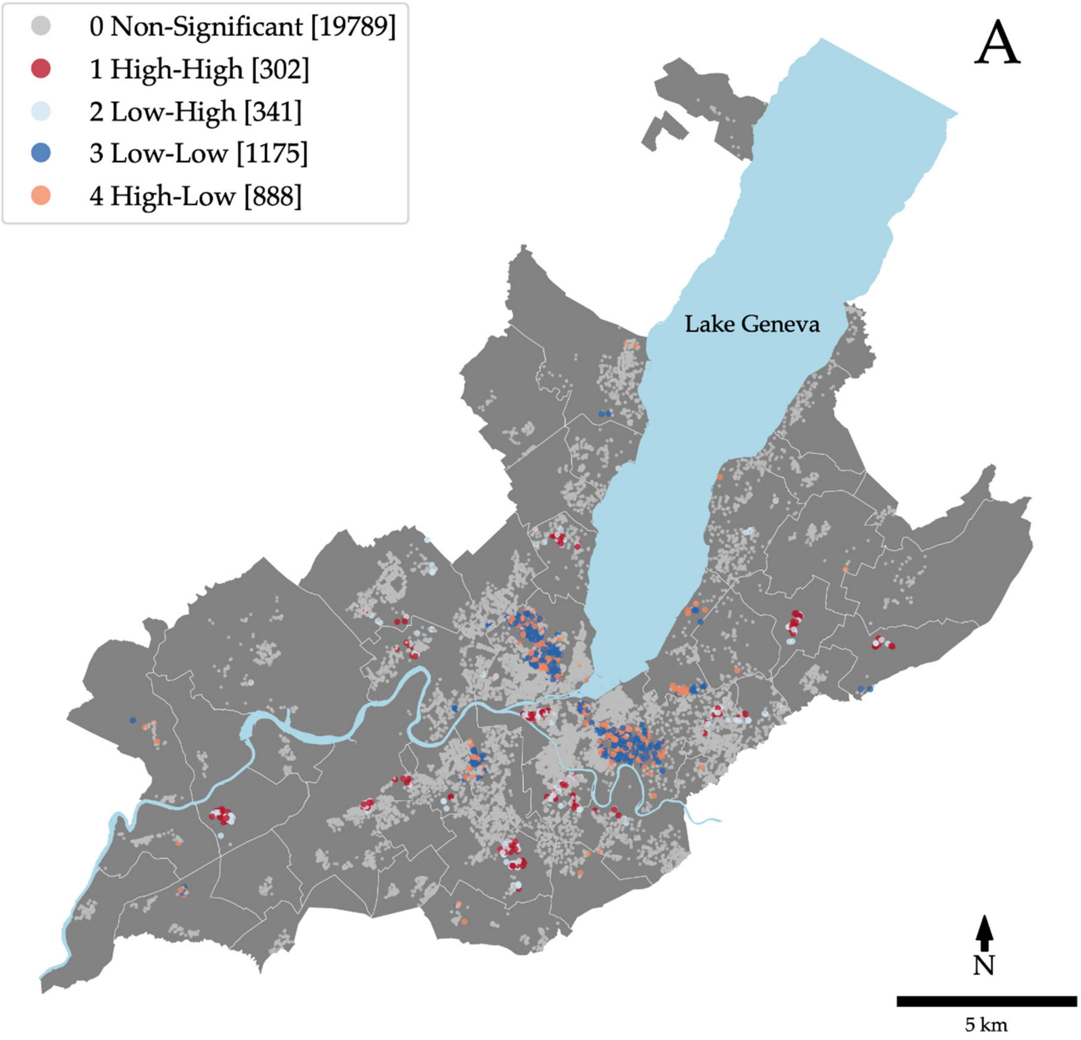

0 Non-Significant [19789]
1 High-High [302]
2 Low-High [341]
3 Low-Low [1175]
4 High-Low [888]
A
Lake Geneva
N
5 km

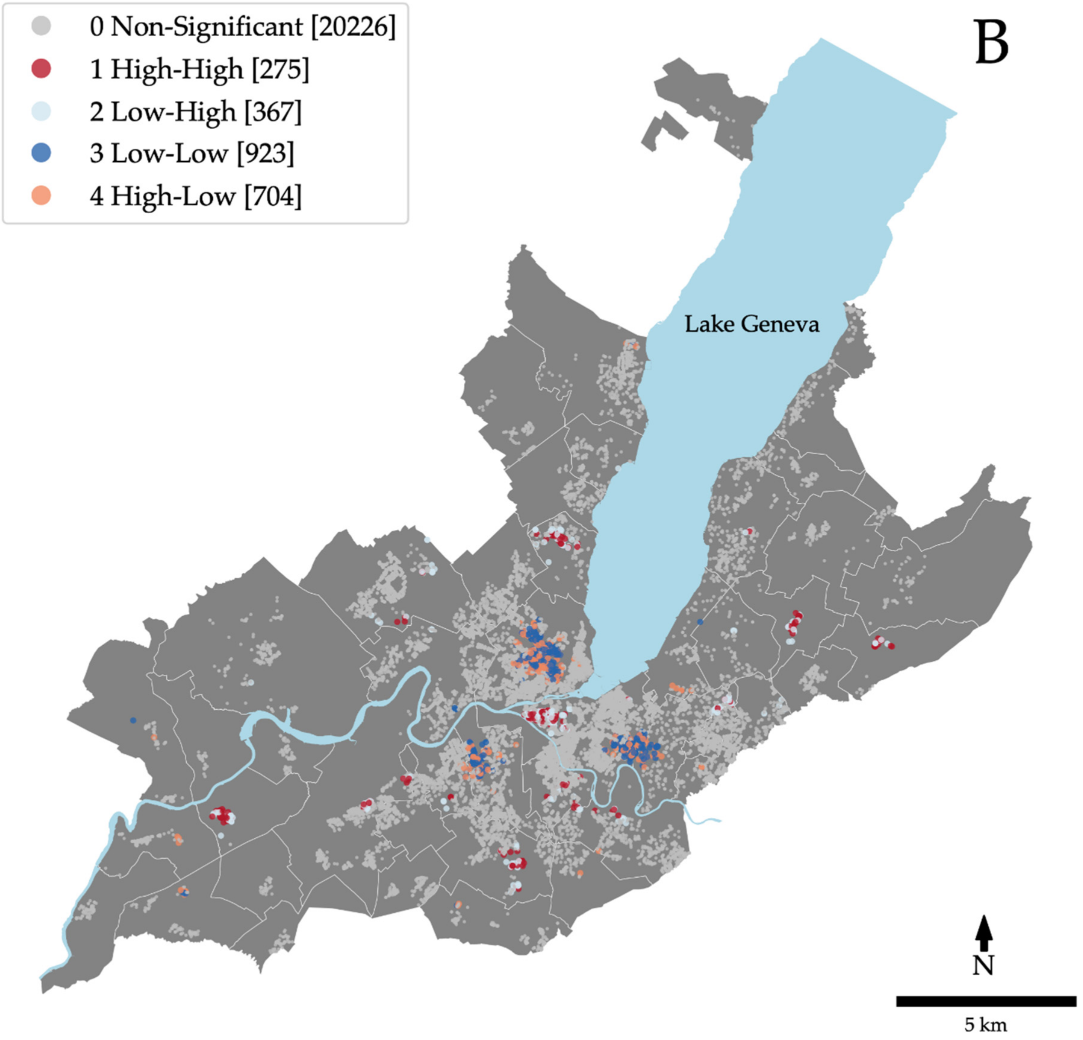

Figure S6. Local Moran's I spatial clusters of Na:K ratio (A) adjusted for food environment characteristics (GWR model 1) and (B) socio-demographic characteristics (GWR model 2) using a geographically weighted regression (GWR). The dark red markers (1 High-High) correspond to the individuals with a high Na:K ratio surrounded by individuals with a high Na:K ratio. The light blue markers (2 Low-High) correspond to individuals with a low Na:K ratio surrounded by individuals with a high Na:K ratio. The dark blue markers (3 Low-Low) correspond to individuals with a low Na:K ratio surrounded by individuals with a low Na:K ratio. The light red markers (4 High-Low) correspond to individuals with a high Na:K ratio surrounded by individuals with a low Na:K ratio. Grey markers are not significant at α = 0.05. White lines correspond to municipality delimitations.

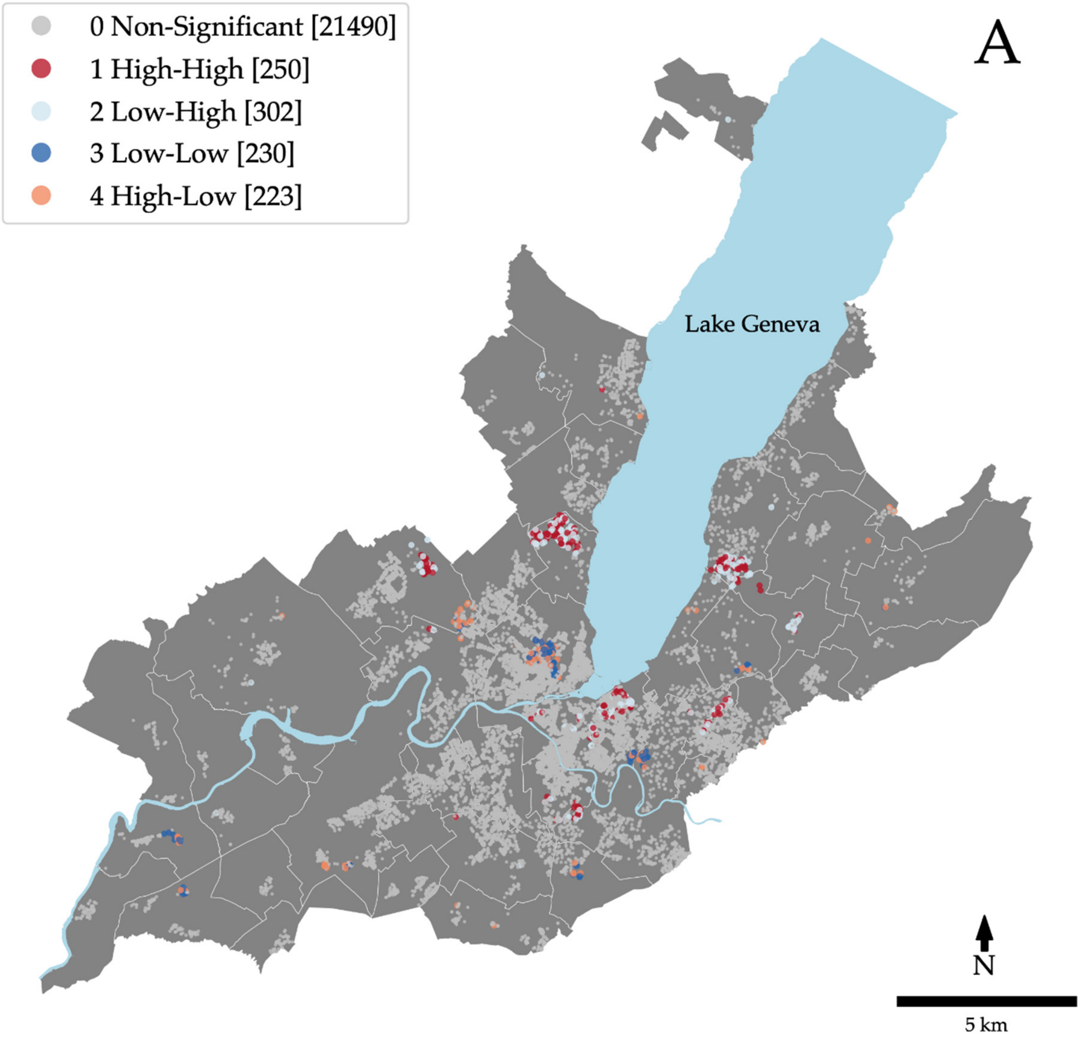
0 Non-Significant [21490]
1 High-High [250]
2 Low-High [302]
3 Low-Low [230]
4 High-Low [223]
A
Lake Geneva
N
5 km

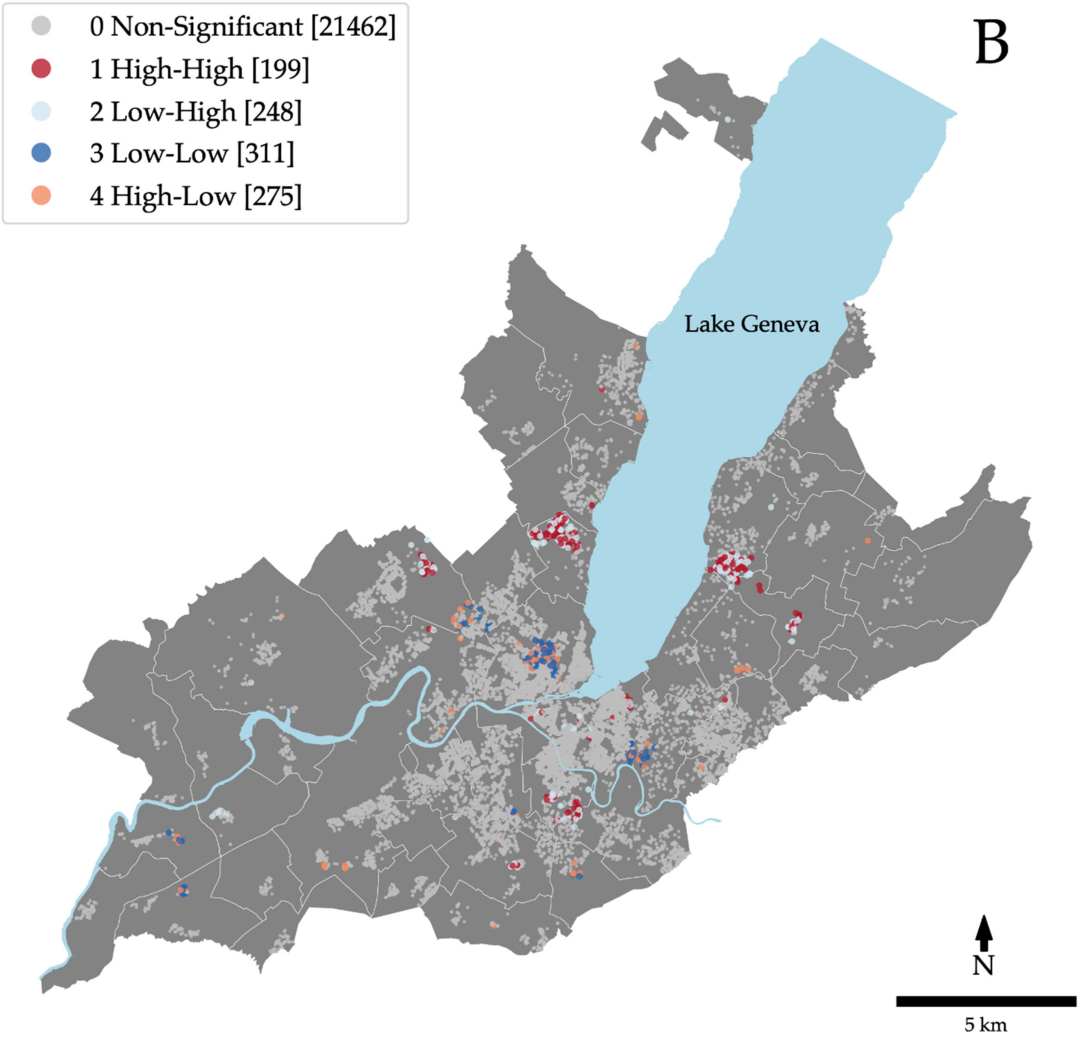

Figure S7. Local Moran's I spatial clusters of Na intake (A) adjusted for food environment characteristics (GWR model 1) and (B) socio-demographic characteristics (GWR model 2) using a geographically weighted regression (GWR). The dark red markers (1 High-High) correspond to the individuals with a high Na intake surrounded by individuals with a high Na intake. The light blue markers (2 Low-High) correspond to individuals with a low Na intake surrounded by individuals with a high Na intake. The dark blue markers (3 Low-Low) correspond to individuals with a low Na intake surrounded by individuals with a low Na intake. The light red markers (4 High-Low) correspond to individuals with a high Na intake surrounded by individuals with a low Na intake. Grey markers are not significant at $\alpha = 0.05$. Black lines correspond to municipality delimitations.

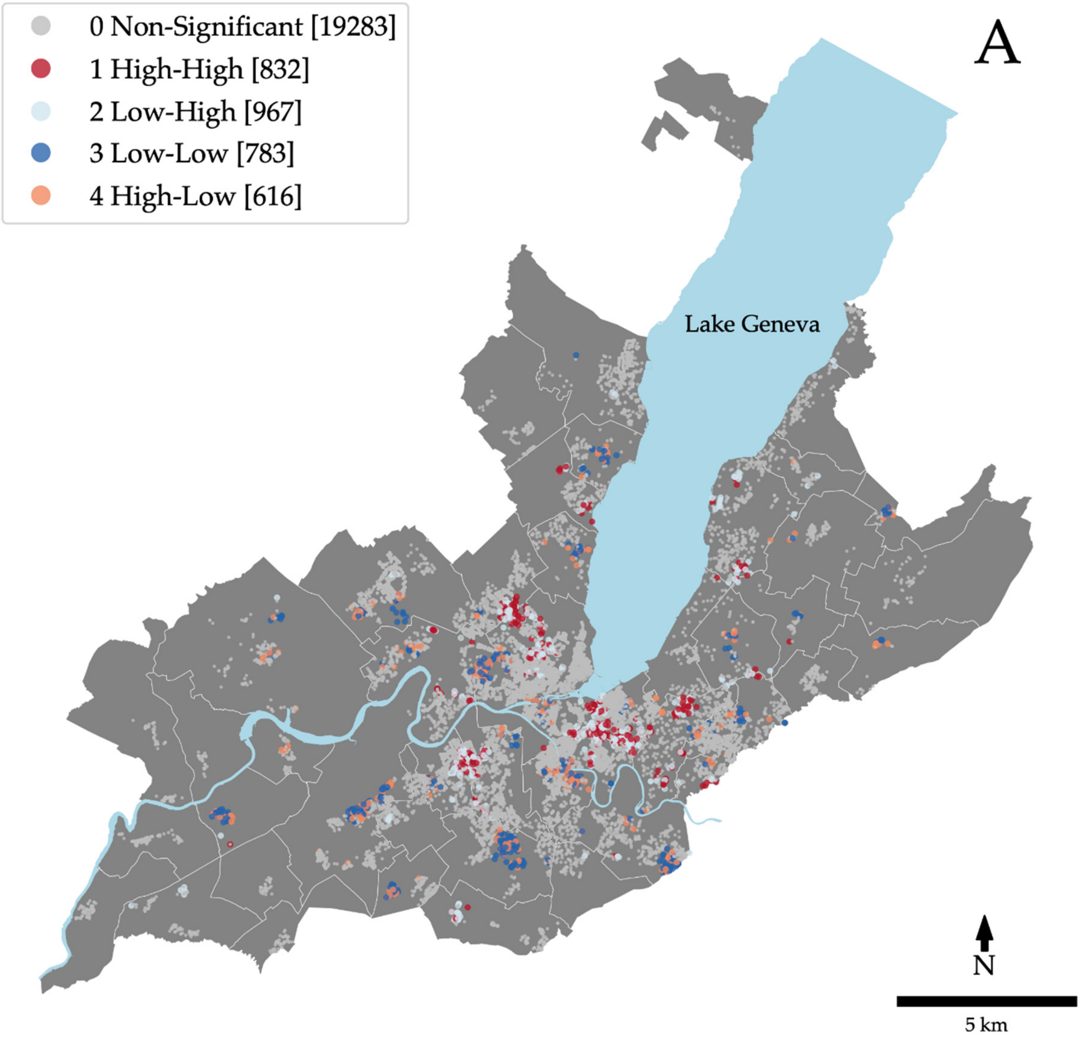
0 Non-Significant [19283]
1 High-High [832]
2 Low-High [967]
3 Low-Low [783]
4 High-Low [616]
A
Lake Geneva
N
5 km

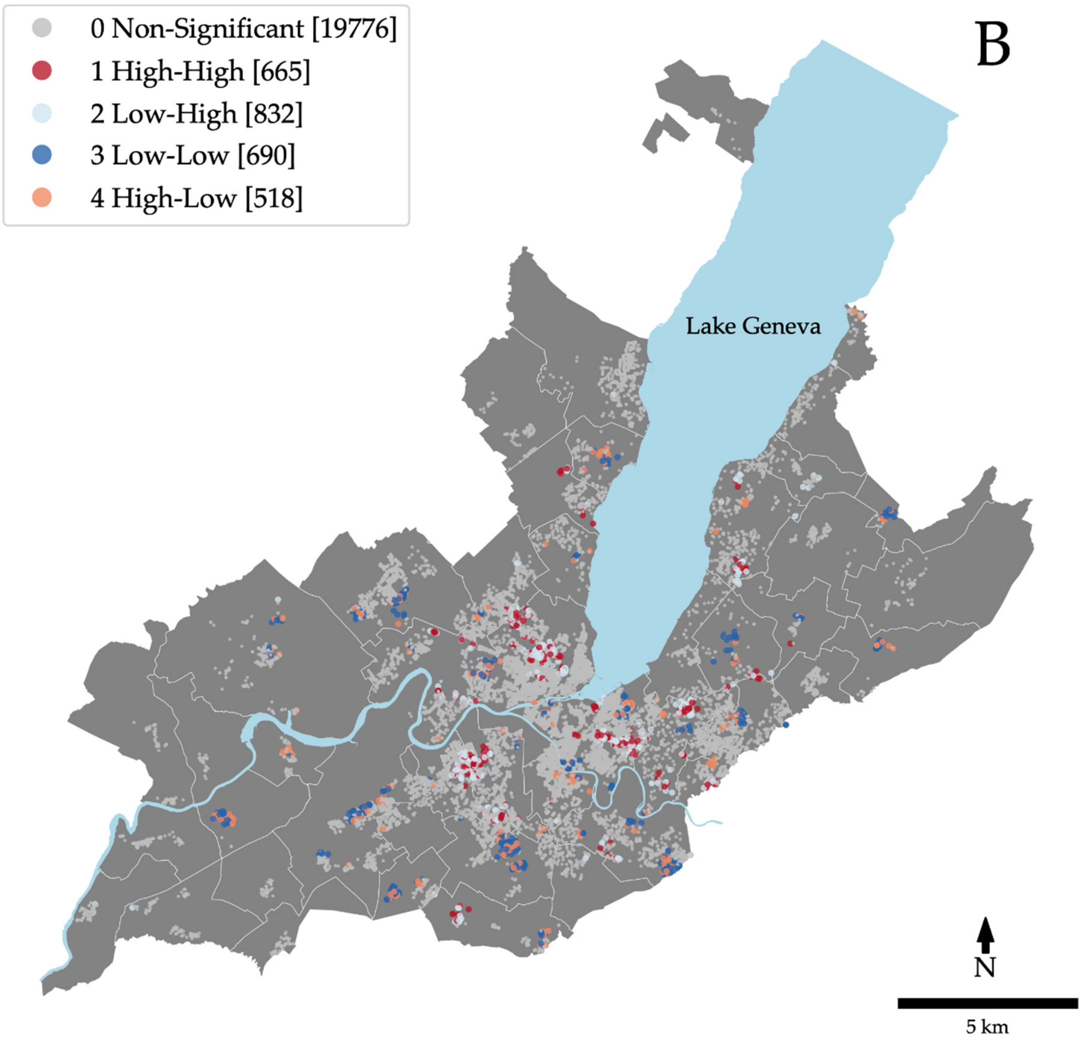

Figure S8. Local Moran's I spatial clusters of K intake (A) adjusted for food environment characteristics (GWR model 1) and (B) socio-demographic characteristics (GWR model 2) using a geographically weighted regression (GWR). The dark red markers (1 High-High) correspond to the individuals with a high K intake surrounded by individuals with a high K intake. The light blue markers (2 Low-High) correspond to individuals with a low K intake surrounded by individuals with a high K intake. The dark blue markers (3 Low-Low) correspond to individuals with a low K intake surrounded by individuals with a low K intake. The light red markers (4 High-Low) correspond to individuals with a high K intake surrounded by individuals with a low K intake. White markers are not significant at $\alpha = 0.05$. Black lines correspond to municipality delimitations.

Table S1. Number of food outlets by period and food outlet category. The periods of participation to the Bus Santé study of each participant are matched to the closest year available in the food outlet dataset.

REG dataset (year)	Periods of participation to the Bus Santé study	Supermarkets (N)	Grocery stores (N)	Convenience stores (N)
2003	1993-2003	43	79	280
2005	2004-2006	66	85	255
2008	2007-2009	67	112	269
2011	2010-2012	56	136	252
2014	2013-2015	130	372	552
2017	2016-2018	148	438	524

Table S2. Global modeling (OLS) of the associations between socio-demographic and food environment characteristics, and Na:K ratio, Na and K intakes (n = 22,495), Bus santé study, Geneva, Switzerland, 1993-2018.

	Na:K Ratio			Na Intake			K Intake		
	**Model 1**	**Model 2**	**Model 3**	**Model 1**	**Model 2**	**Model 3**	**Model 1**	**Model 2**	**Model 3**
	β, 95% CI	β, 95% CI	β, 95% CI	β, 95% CI	β, 95% CI	β, 95% CI	β, 95% CI	β, 95% CI	β, 95% CI
Intercept	1.43 ***, [1.42, 1.43]	1.49 ***, [1.45, 1.53]	1.49 ***, [1.45, 1.53]	3743.29 ***, [3731.28, 3755.30]	3759.36 ***, [3683.64, 3835.07]	3759.87 ***, [3684.13, 3835.61]	2723.90 ***, [2714.94, 2732.86]	2640.87 ***, [2586.62, 2695.12]	2639.89 ***, [2585.63, 2694.16]
Total Energy intake	0.11 ***, [0.10, 0.12]	0.10 ***, [0.09, 0.10]	0.10 ***, [0.09, 0.10]	1322.11 ***, [1310.02, 1334.21]	1347.20 ***, [1334.58, 1359.83]	1347.27 ***, [1334.64, 1359.89]	750.83 ***, [741.81, 759.85]	804.77 ***, [795.73, 813.82]	804.76 ***, [795.72, 813.81]
Year of survey	-0.04 ***, [-0.05, -0.03]	-0.04 ***, [-0.05, -0.03]	-0.04 ***, [-0.05, -0.03]	-32.82 ***, [-45.73, -19.92]	-33.24 ***, [-46.60, -19.89]	-31.62 ***, [-45.79, -17.45]	63.25 ***, [53.62, 72.88]	66.48 ***, [56.91, 76.05]	64.73 ***, [54.58, 74.88]
Age		-0.02 ***, [-0.03, -0.02]	-0.02 ***, [-0.03, -0.02]		49.11 ***, [36.70, 61.53]	49.06 ***, [36.63, 61.49]		79.27 ***, [70.38, 88.17]	79.42 ***, [70.51, 88.32]
Gender		-0.11 ***, [-0.12, -0.10]	-0.11 ***, [-0.12, -0.10]		191.72 ***, [165.68, 217.76]	191.73 ***, [165.69, 217.77]		380.87 ***, [362.21, 399.53]	380.89 ***, [362.23, 399.55]
Nationality		0.05 ***, [0.04, 0.07]	0.05 ***, [0.04, 0.07]		-66.48 ***, [-93.42, -39.54]	-66.82 ***, [-93.81, -39.83]		-161.73 ***, [-181.03, -142.43]	-160.84 ***, [-180.18, -141.50]
High skilled occupation		0, [-0.03, 0.04]	0, [-0.03, 0.04]		-34.25, [-102.37, 33.87]	-34.46, [-102.62, 33.69]		-36.82, [-85.63, 11.99]	-37.21, [-86.04, 11.62]
Medium skilled occupation		-0.01, [-0.04, 0.03]	-0.01, [-0.04, 0.03]		-76.51 *, [-141.10, -11.92]	-76.62 *, [-141.22, -12.01]		-35.14, [-81.42, 11.14]	-35.21, [-81.50, 11.08]
Low skilled occupation		-0.02, [-0.05, 0.02]	-0.02, [-0.05, 0.02]		-55.65, [-120.52, 9.23]	-55.94, [-120.85, 8.96]		-6.48, [-52.97, 40.00]	-6.7, [-53.20, 39.80]
Tertiary education		-0.06 ***, [-0.08, -0.03]	-0.06 ***, [-0.08, -0.03]		-3.86, [-44.20, 36.48]	-4.17, [-44.58, 36.24]		85.35 ***, [56.45, 114.26]	84.85 ***, [55.90, 113.80]
Secondary education		-0.02, [-0.04, 0.00]	-0.02, [-0.04, 0.00]		-11.22, [-49.71, 27.27]	-11.32, [-49.81, 27.17]		20.03, [-7.54, 47.61]	20.07, [-7.50, 47.65]
Neighborhood median income		0, [-0.01, 0.01]	0, [-0.01, 0.01]		-0.41, [-12.97, 12.15]	-0.83, [-14.46, 12.80]		-2.74, [-11.74, 6.26]	-0.47, [-10.24, 9.30]
Married		0, [-0.02, 0.01]	0, [-0.02, 0.01]		-3.92, [-31.34, 23.50]	-3.79, [-31.39, 23.80]		-22.30 *, [-41.95, -2.65]	-21.29 *, [-41.06, -1.52]
Convenience store density (800m)	0.01, [-0.00, 0.02]		0.01, [-0.00, 0.02]	11.33, [-6.95, 29.61]		10.55, [-7.90, 29.00]	-3.15, [-16.79, 10.49]		-5.88, [-19.09, 7.34]
Grocery store density (800m)	0, [-0.01, 0.01]		0, [-0.01, 0.01]	-6.52, [-23.39, 10.34]		-4.43, [-21.32, 12.46]	1.46, [-11.12, 14.04]		4.1, [-8.01, 16.20]
Supermarket density (800m)	-0.01 *, [-0.02, -0.00]		-0.01 *, [-0.02, -0.00]	-8.82, [-23.28, 5.64]		-10.27, [-24.76, 4.21]	12.77 *, [1.98, 23.56]		10.96 *, [0.58, 21.34]
AIC	31529.79	31157.7	31159.35	370828.28	370556.68	370560.41	357643.29	355557.15	355558.34
Moran's *I*	0.0009*	0.0005	0.0004	-0.0003	-0.0004	-0.0004	0.003***	0.001*	0.0009*

All continuous predictors are mean-centered and scaled by 1 standard deviation. *** $p < 0.001$; ** $p < 0.01$; * $p < 0.05$.

Table S3. Local modeling (GWR) of the associations between socio-demographic and food environment characteristics, and Na:K ratio, Na and K intakes (n = 22,495), Bus santé study, Geneva, Switzerland, 1993-2018.

Geographically Weighted Regression Analysis

	**Na:K Ratio**			**Na Intake**			**K Intake**		
	**Model 1**	**Model 2**	**Model 3**	**Model 1**	**Model 2**	**Model 3**	**Model 1**	**Model 2**	**Model 3**
	β[a]	β[a]	β[a]	β[a]	β[a]	β[a]	β[a]	β[a]	β[a]
Intercept	1.415, 1.588	1.417, 1.438	1.420, 1.459	3722.024, 3765.197	3727.956, 3761.301	3725.783, 3776.973	2550.117, 2747.296	2686.493, 2739,224	2697.614, 2736.854
Total energy intake	0.104, 0.157	0.088, 0.0148	0.088, 0.0148	1319.370, 1394,362	1344.669, 1429.279	1309.044, 1423.712	670.435, 765.868	739.672, 811.808	741.619, 811.815
Year of survey	-0.062, -0.001	-0.053, -0.031	-0.055, -0.029	-64.415, -5.414	-47.516, -15.938	-46.759, -12.981	-17.923, 105.865	40.319, 77.506	41.865, 76.901
Age		-0.048, -0.004	0.048, -0.004		11.478, 60.259	15.187, 59.525		6.320, 105.427	6.806, 104.836
Gender		-0.064, -0.028	-0.063, -0.029		81.661, 108.306	83.437, 105.845		150.765, 199.506	151.486, 199.527
Nationality		0.015, 0.042	0.015, 0.042		-49.371, -0.844	-47.696, -0.631		-104.456, -62.260	-102.891, -62.024
High skilled occupation		-0.034, 0.102	-0.035, 0.101		-30.539, 130.989	-29.871, 116.617		-112.804, 36.475	-112.845, 38.229
Medium skilled occupation		-0.079, 0.071	-0.079, 0.071		-105.972, 89.427	-99.844, 79.374		-100.283, 50.274	-100.157, 52.355
Low skilled occupation		-0.072, 0.072	-0.072, 0.072		-103.256, 105.758	-97.931, 92.995		-94.831, 34.477	-94.181, 36.479
Tertiary education		-0.085, -0.009	-0.086, -0.011		-46.580, 8.390	-43.517, 5.765		12.731, 124.853	12.231, 124.477
Secondary education		-0.059, -0.013	-0.059, 0.013		-59.808, 41.460	-55.849, 36.900		-22.440, 69.745	-22.186, 68.815
Neighborhood median income		-0.011, 0.020	-0.017, 0.015		-24.140, 14.805	-28.667, 13.038		-41.197, 3.492	-34.567, 6.407
Married		-0.041, 0.003	-0.041, 0.003		-72.187,5.996	-64.343, 5.235		-12.741, 15.641	-12.817, 14.793
Convenience store density (800m)	-0.108, 0.207		-0.049, 0.049	-3.127, 77.141		-15.416, 60.776	-29.119, 244.316		-10.131, 70.653
Grocery store density (800m)	-0.011, 0.136		-0.013, 0.059	-18.492, 45.108		-24.138, 39.827	-417.754, 13.955		-139.964, 6.887
Supermarket density (800m)	-0.036, 0.001		-0.024, -0.003	-25.580, -5.568		-25.253, -6.501	-16.211, 89.886		2.613, 36.089
AICc optimized bandwidth (m)	3590.68	4071.22	4071.18	4551.780	4368.2	4551.78	2516.17	4071.22	4071.22
AICc	31521.791	31143.107	31143.119	370820.72	370545.79	370549.49	357610.031	355545.341	355546.013
Moran's *I*	0.0005	0.0003	0.0001	-0.0004	-0.0005	-0.0006	0.0007	0.0004	0.0003

[a], Range of locally estimated coefficients

Table S4. Global Moran's *I* statistics, z-scores and *p*-values calculated incrementally at a fixed distance band of 200m, 400m, 600m, 800m and 1000m.

Variable	Distance (m)	Global Moran's I	*p*-value	z-score
**Na:K Ratio**	200	0.002	0.140	1.124
	400	0.000	0.298	0.517
	600	0.001	0.131	1.179
	**800**	**0.001**	**0.047**	**1.747**
	1000	0.001	0.117	1.179
**Na Intake**	200	0.001	0.324	0.454
	400	0.000	0.440	0.110
	600	0.000	0.332	0.411
	800	0.000	0.441	-0.128
	1000	0.000	0.436	-0.180
**K Intake**	200	0.003	0.034	1.864
	**400**	**0.002**	**0.036**	**1.929**
	600	0.001	0.053	1.807
	800	0.000	0.245	0.723
	1000	0.001	0.145	1.039
